# Integration of psychological interventions in multi-sectoral humanitarian programmes: a systematic review

**DOI:** 10.1186/s12913-024-11704-7

**Published:** 2024-12-02

**Authors:** Jacqueline N. Ndlovu, Jonna Lind, Andrés Barrera Patlán, Nawaraj Upadhaya, Marx R. Leku, Josephine Akellot, Morten Skovdal, Jura L. Augustinavicius, Wietse A. Tol

**Affiliations:** 1 Global Health Section, University of Copenhagen, Øster Farimagsgade 5, Building 9, Copenhagen, 1353 Denmark; 2 ARQ Library, ARQ National Psychotrauma Centre, Diemen, The Netherlands; 3 Department of Epidemiology, Biostatistics, and Occupational Health, Faculty of Medicine and Health Sciences, McGill University, Montreal, Canada; 4 HealthRight International, New York, USA; 5 Section of Health Services Research, University of Copenhagen, Copenhagen, Denmark; 6 School of Population and Global Health, Faculty of Medicine and Health Sciences, McGill University, Montreal, Canada; 7 Athena Research Institute, Vrije Universiteit Amsterdam, Arq International, Diemen, the Netherlands

**Keywords:** Psychological interventions, Multi-sectorial, Integration, Humanitarian settings

## Abstract

**Background:**

Every year, millions of people are affected by humanitarian crises. With a growing population of people affected, the need for coordination and integration of services aiming to improve the effectiveness of mental health and psychosocial support also grows. In this study, we examine how psychological interventions in humanitarian settings globally have been implemented through integration into programming outside of formal healthcare delivery through multisectoral integration.

**Methods:**

A comprehensive search of six databases and reference checking was undertaken in 2022. We included studies focusing on implementation strategies and implementation outcomes of multi-sectoral, integrated psychological interventions, with no year limits. We extracted data using the software Covidence, and used the software to manage screening and reviewing processes. All studies were critically appraised for quality and rigor using the mixed-methods appraisal tool.

**Results:**

Eight studies were included in total. We found that interventions targeted conflict affected, displaced and disaster recovering populations. The interventions demonstrated moderate success in reducing psychological distress and enhancing disaster preparedness. We found that key implementation outcomes investigated and prioritised include acceptability, feasibility, and relevance. The studies reported on integration processes that involved task shifting primarily, with an emphasis on different formats of adaptation, partnership creation and capacity development to maximise effectiveness of integrated interventions.

**Conclusion:**

Overall, there is little research being done to rigorously document the processes and experiences of integrating psychological interventions with non-health interventions. This could be an indication that, while multisectoral integration may be more common in practice, little research is being done or reported in this area formally. There is an urgent need for further research into integrated multi-sectoral interventions. This research should aim to understand how social, cultural, and environmental contexts in different ways, and to different degrees, affect what is acceptable and feasible to deliver and how these ultimately influence the impact of integrated interventions.

**Supplementary Information:**

The online version contains supplementary material available at 10.1186/s12913-024-11704-7.

## Background

Every year, millions of people are affected by humanitarian crises [1]. Humanitarian crises, including disasters, conflicts and political unrest, lead to widespread displacement, increased mortality and morbidity and long-term socio-economic disruptions. There is a growing body of research that documents the psychological distress and social suffering faced by people affected by humanitarian crises [2]. More specifically, one in every five people meets the criteria for moderate/severe mental health conditions such as depression, anxiety, post-traumatic stress disorder (PTSD), bipolar disorder or schizophrenia [2]. In addition to psychological distress, people affected by humanitarian crises face challenges accessing food, water, shelter, health care, education, social protection and employment opportunities among others. Consequently, there is a great need for responsive mental health and psychosocial support (MHPSS) activities that address the multifaceted needs of individuals affected by humanitarian crises.

The term MHPSS, adopted by the Inter Agency Standing Committee (IASC) in 2007, is an umbrella term that broadly includes “any type of local or outside support that aims to protect or promote psychosocial wellbeing and prevent or treat mental disorders” [3]. Meeting mental health needs of populations in a context of social upheaval and resource scarcity is challenging in many cases [4, 5]. People affected by humanitarian crises are likely to have fewer psychological and social resources available to help cope with stressful events compared to the general population. Also, individuals experiencing mental health challenges often do not receive the support that they need [6]. Mental health and wellbeing are determined by multiple, interrelated social and environmental factors [7]. Thus, people will have different types of needs that need to be met; from ensuring that basic needs are appropriately met, to providing specialised mental health care. Consensus-based guidelines recommend the implementation of multi-layered care (providing diverse and integrated support levels, from basic services to specialized mental health care) to meet these varied needs.

People are affected by humanitarian crises in different ways, which is why multi-layered care systems are more ideal for addressing challenges faced. Thus, more attention has been drawn to synergise activities between sectors so as to holistically address challenges, foster sustainable MHPSS responses and achieve greater impact [8]. A recent consensus-based research agenda for MHPSS highly prioritised research on the integration of MHPSS across diverse sectors [9]. Despite this, siloed implementation of interventions, where implementation only addresses mental health challenges and not the associated social and environmental determinants, appears to be common. Siloed implementation may be very costly and, in many cases, can exacerbate the already limited funding for and reach of interventions [10]. When MHPSS is implemented across different sectors, difficulties such as insufficient resources for effective implementation and unclear roles within programme implementation, lead to non-participation of stakeholders [11]. This problem is not unique to humanitarian crises - it is also observed in other areas of public health [12–14]. Multisectoral integration has been recommended as an ideal strategy to address identified issues in a more holistic manner and aligns with a growing interest and need to reach more people and close the treatment gap [15]. However, there are few examples of multi-sectoral integration outside of the health sector.

This review seeks to provide a focused understanding of multi-sectoral integration outside of the health sector in humanitarian settings. We have limited focus to a small subset of MHPSS and focus only on psychological interventions. Within the purposefully broad MHPSS term, psychological interventions exist as a subcategory in this context. Psychological interventions include a broad range of treatments and therapies designed to help individuals experiencing psychological and emotional distress overcome challenges and improve overall wellbeing [16]. These interventions can be delivered in many ways, such as in individual or group settings, in counselling structures, or tailored to meet the specific needs of populations [16]. By delimiting the scope to psychological interventions, the review can deeply delve into the nuances of psychological interventions and provide a more comprehensive grasp of specific interventions and their associated mechanisms of action. Focusing solely on psychological interventions in our systematic review allows for a detailed, resource-efficient, and evidence-based analysis, ensuring measurable outcomes and addressing the most pressing needs directly. Thus, the review seeks to contribute insights, perspectives and methodologies inspired by psychological interventions, that may complement and enrich discussions within the broader field of MHPSS.

Additionally, the review specifically focuses on psychological intervention integration into non-health care sectors. This is a relatively unexplored area, as previous literature has described integrating psychological interventions into various health care systems (e.g., primary care, maternal health care, HIV/AIDS care) [17]. There are several reasons why integrating psychological interventions into non-health care sectors may be a critical and complementary strategy to integrating them into health care sectors. *First*, integrating psychological interventions in non-health care sectors can help address social determinants of mental health at the same time. Integrating psychological interventions in non-healthcare sectors such as education, livelihoods and protection programming is an important step in contributing to reducing mental health inequalities based on differential exposure to adverse social, economic, and environmental conditions [18–20]. *Second*, addressing social determinants can also save costs, as costs associated with mental health challenges are borne outside of the healthcare sector. For example, more resources are spent on social security programmes such as unemployment benefits and sick leave benefits due to mental health challenges faced by people, and addressing mental health will also therefore reduce social spending [21]. *Third*, in humanitarian settings, it might not be possible to add on services to already weak and unstable healthcare systems. *Fourth*, integration may also be a strategy to facilitate the expansion of MHPSS beyond the health sector, with greater potential for reaching more people in a less stigmatising environment.

This review aims to examine how psychological interventions in humanitarian crisis settings globally have been implemented through integration into programming outside of formal healthcare delivery, across various humanitarian sectors. The review scope builds on a study on barriers and facilitators for scaling up MHPSS interventions for populations affected by humanitarian crises [17]. As this topic is under researched, there is limited knowledge on implementation processes for different population groups. We chose to focus this review on adults to get a better understanding of this specific population group as there is a research gap. Research on integrating mental health interventions in school settings for children is more advanced than with adults, and implementation processes may potentially be different [22, 23]. For the purposes of this review, programming outside of formal and specialised health care will be referred to as non-health sectors. Specifically, the review will (a) identify implementation strategies and implementation outcomes used in integrating psychological interventions in multi-sectorial humanitarian programming globally; and (b) assess good implementation practices and challenges of integrating psychological interventions across sectors outside of health for people at risk of, or experiencing, psychological distress and/or common mental disorders. Despite multisectoral, integrated approaches to mental health having been long recommended, to the best of our knowledge, there has been no systematic synthesis of literature on the integration of psychological interventions into non-health sectors in humanitarian settings.

## Methods

### Protocol, registration, reporting guidelines

The protocol was registered with the National Institute for Health Research’s International prospective register of systematic reviews (PROSPERO 2022 CRD42022316223). This systematic review was conducted using the preferred reporting items for systematic review (PRISMA) updated guidelines [24]. The PRISMA Checklist is included in Additional file 1.

### Operationalisation of key terms

The development of MHPSS as a standardised term was initiated to provide a comprehensive framework for addressing the mental health and psychosocial support needs of individuals and communities. It helped to facilitate communication and coordination among different humanitarian actors, organisations and sectors involved in emergency and humanitarian response efforts. Unfortunately, there are still many terms and concepts in this field that are used differently across sectors. In this review, we operationalise some of these terms and concepts central to the review by defining them based on literature and the specific context of our study (Table 1).

**Table 1:** Overview of operationalised key terms related to the review

Key term	Definition	Operational definition
MHPSS interventions	Range of structured activities, programmes, and services designed to address MHPSS needs of individuals and communities affected by humanitarian crises. These interventions include both mental health-focused interventions (e.g., psychotherapy, counselling) and psychosocial support activities (e.g., group support, community-based interventions) aimed at promoting resilience, coping skills, and social connectedness [25]	Two parts: *(i) Mental health-focused* interventions including individual or group-based therapies, e.g. cognitive-behavioural therapy (CBT) *(ii) Psychosocial support* activities encompassing non-clinical interventions aimed at addressing psychosocial needs and fostering social support networks, such as community support groups and peer support programmes Both are direct and indirect interventions, delivered by trained professionals, lay counsellors, community volunteers, or peer facilitators
Psychological interventions	Refers to a wide range of therapies, techniques, and strategies designed to address mental health issues, promote psychological well-being, and improve overall quality of life. These interventions are delivered by trained personnel and can be tailored to meet the specific needs of individuals or groups	Aim to address psychological distress, improve mental health and promote overall well-being through targeted strategies and techniques Indicators: delivered by trained personnel, focus on mental processes or behaviour, based on psychological principles
Multi-sectoral integration	Involves bringing together professionals, organisations, and stakeholders from diverse sectors (health, education, social services, humanitarian aid, government agencies, and NGOs). Stakeholders work collaboratively to design, implement, and evaluate psychological interventions that address the full spectrum of needs [26]	Collaboration and coordinated service delivery between key sectors Provision of integrated services that address both mental health needs of individuals and communities and other essential services such as healthcare, education, and social support Indicators: existence of formal partnerships or agreements between sectors, establishment of joint planning and decision-making mechanisms, and the integration of psychological intervention components into broader humanitarian programmes
Non-health sector	Programming outside of formal and specialised health care such as humanitarian clusters (protection, education, water sanitation hygiene, shelter, food security, logistics, emergency telecommunications, nutrition, early recovery, camp coordination and camp management) [27]	Includes community-based interventions using non-clinical approaches Use of diverse service providers and stakeholders involved in mental health promotion and support outside of health, such as community organisations, grassroots initiatives, peer support networks and faith-based organisations Addresses the social determinants of mental health, promotes community participation and empowerment, and recognises the importance of cultural and contextual factors

### Eligibility criteria

We included studies focusing on implementation strategies and implementation outcomes of multi-sectoral, integrated psychological interventions. We also included all types of studies (quantitative, qualitative and mixed-methods) that described the implementation of psychological interventions in humanitarian settings through integration with programming in other (non-health) sectors (referred to as multi-sectoral integration). Studies that describe the implementation of psychological interventions in health-care settings (e.g., primary care, maternal and child healthcare, HIV care and mental health care) were excluded. Studies reporting on original data regarding implementation processes concerning multi-sectoral integration were included. Implementation strategies in this review refer to methods or techniques that are used to strengthen adoption, implementation and sustainability of evidence-based interventions [28]. Due to challenges and inconsistencies in specifying and reporting implementation strategies, we used a framework developed by Proctor and colleagues which outlines prerequisites to measuring implementation strategies [28]. These include feasibility, acceptability, adoption, relevance, fidelity, sustainability, implementation costs and penetration. In addition, we define multi-sectoral integration in this context as the use of psychological interventions outside of the health sector or cluster. Studies were included if they engaged with adults aged 18 years and older who were experiencing, or at risk of experiencing, psychological distress and/or common mental disorders in humanitarian settings globally. These included adults who were going through or had been exposed to disasters and armed conflict settings. A table detailing all inclusion and exclusion criteria is included in Addition file 2. We have also included examples of studies excluded for reference (Additional file 3).

### Information sources

On February 16 2022, the following six databases were searched: Ovid Medline ALL (Ovid, 1946-…), PsycInfo (Ovid, 1806-…), Embase (Ovid, 1974-…), Ovid Evidence-Based Medicine Reviews (Ovid - Cochrane DSR 2005-…, ACP Journal Club 1991-…, DARE 1991–2015, CCA 2012-…, CCTR 1991-…, CMR 1995–2012, HTA 2001–2016, and NHSEED 1995–2015) and PTSDpubs (National Center for PTSD, VS; 1871-…). Some authors of studies included in the review were contacted as well to find out if there had been any further developments in their research that we could include in our study. We also searched references cited in systematic reviews identified on similar topics and reference lists of studies included in this review.

### Search strategy

The search strategy was a modification and expansion of the one used by Troup and colleagues [28]. We first built a search strategy in Ovid Medline ALL, which we translated and adapted to the other databases. The full search strategies are included in Additional file 4.

The search terms were grouped into four main clusters. Terms were related to (i) populations (people at risk of/or experiencing psychological distress and common mental disorders (CMDs) (i.e., depression, anxiety, PTSD, and somatoform conditions), (ii) interest (psychological interventions, integration, non-health sectors), (iii) context (humanitarian settings globally), and (iv) methodology i.e., qualitative, quantitative or mixed method studies. We also searched for aggregated evidence in reviews to be able to use their bibliographies for more studies. These clusters were combined using Boolean operators (and, or).

### Selection process

We used the software Covidence to manage screening, reviewing and extraction processes for this review in two phases [29]. First, two authors (JNN and ABP) independently screened all titles and abstracts to identify studies that met the inclusion criteria through double screening. Secondly, the same authors then independently screened the full texts of the included studies in greater depth against the inclusion and exclusion criteria. We worked in an iterative way where publications meeting all inclusion criteria were included, and publications meeting one or more exclusion criteria were excluded, citing the first exclusion criterion that was met. Studies that were identified as not relevant by both authors were excluded without review. Conflicts were resolved through dialogue and the help of co-authors when consensus was not reached.

### Data collection process

The following data were extracted into an Excel spreadsheet and cross-checked by one of the research team members for accuracy: (1) author and publication year; (2) country where the study was conducted; (3) psychological intervention type based on descriptions provided in the studies; (4) psychological distress or common mental disorders such as anxiety, depression and PTSD; (5) type of service providers (description of those implementing the integrated interventions) and overall intervention description; (6 and 7) implementation outcomes and implementation strategies as described by Proctor and colleagues [28]; (8) the non-health sector and interventions within which psychological interventions have been integrated; (9) description of integration processes; (10) target impact group and type of humanitarian setting; (11) key reported facilitators to integration; and (12) key reported challenges related to the integration and implementation processes (Table 2).

**Table 2:** Study characteristics

Author (year)	Country	Psychological intervention type	Psychological distress/CMD	Service providers and intervention	Implementation outcome	Implementation strategies	Non-health integration and sector	Integration process	Target impact group and humanitarian crises	Key facilitators reported	Key challenges reported
Greene et al. (2019)	Tanzania	Integrated elements of Advocacy Counselling and Cognitive Processing Therapy (CPT)	Stress, too many thoughts, deep sadness, fear	Lay refugee incentive workers delivering a multi-sectoral integrated violence and mental health focused intervention (Nguvu)	Relevance Acceptability Feasibility	Shortened version of CPT and advocacy counselling to promote feasibility Task shifting Adaptations Training and supervision	Intimate partner violence (IPV) Protection sector	Learning how to integrate CPT and advocacy counselling with IPV intervention through: (i) Desk review (ii) Site visits (iii) Formative qualitative research (iv) Expert consultations	Congolese refugee women from Nyarugusu refugee camp in Tanzania who have fled from conflict in the eastern provinces of the Democratic Republic of Congo (DRC)	(i) Lay refugee incentive workers trained well in delivering the integrated intervention	(i) Lower rates of intervention and session attendance among women experiencing more severe IPV (ii) Modification of CPT might have contributed to smaller effect sizes for mental health outcomes (iii) Inconsistent quality in delivering the integrated intervention by facilitators
Greene et al. (2022)	Tanzania	Integrated elements of Advocacy Counselling and Cognitive Processing Therapy (CPT)	Stress, too many thoughts, deep sadness, fear	Lay refugee incentive workers delivering a multi-sectoral integrated violence and mental health focused intervention (Nguvu)	Relevance Acceptability Feasibility	Shortening intervention to improve feasibility Use of trained non-specialists (task sharing model) Community advisory board formed to guide implementation approach	Intimate partner violence (IPV) Protection sector	Adapted version of CPT (mental health) combined with 2 sessions of advocacy counselling (IPV)	Congolese refugee women in Nyarugusu refugee camp, who had fled conflict in the eastern provinces of the Democratic republic of Congo (DRC)	Qualitative results: (i) Intervention aligned with local needs and filled a gap in mental health and IPV programming (ii) Formatting of the intervention was acceptable as most women were comfortable discussing sensitive topics in a group setting	(i) Some facilitators struggled with more difficult concepts and needed more support (ii) Complexity of the intervention as a multi-sectoral integrated implementation model (iii)Relevance could have been improved by integrating economic empowerment, microfinance and skills training components
Greene et al. (2021)	Tanzania	Integrated elements of Advocacy Counselling and Cognitive Processing Therapy (CPT)	Psychological distress, functional impairment	Lay refugee incentive workers delivering a multi-sectoral integrated violence and mental health focused intervention (Nguvu)	Relevance Acceptability Feasibility	Task shifting model used to enhance feasibility and sustainability	Intimate partner violence (IPV) Protection sector	Adapted version of CPT (mental health) combined with 2 sessions of advocacy counselling (IPV)	Congolese refugee women from Nyarugusu refugee camp in Tanzania who have fled from conflict in the eastern provinces of the Democratic Republic of Congo	(i) Need to shorten CPT from 12 to 6 sessions to increase feasibility (ii) Delivery of the intervention by lay refugee workers was feasible (iii) Local training and supervision could potentially help in sustainability (iv) Intervention content modified based on feedback throughout intervention	(i) The functional impairment measure used might need to be developed for the context, instead of being adapted (ii) Barriers in intervention procedures e.g. communication and coordination
Schafer et al. (2014)	Gaza (Palestine)	Psychological First Aid (PFA)	Distress	Child Friendly Spaces (CFS) facilitators, farmers and livelihoods extension officers and youth mentors implementing an integrated livelihoods and psychosocial support intervention	Acceptability	Implementing staff and partner organisation staff trained in implementing an integrated approach Capacity building of CFS facilitators, farmer and livelihoods extension officers and youth mentors	Livelihoods project focused on integration of psychosocial support activities Food security sector (livelihoods)	Integration of a psychosocial support approach to livelihoods programming	Communities impacted during the 2008/2009 Israeli incursions known as Operation Cast Lead & 2012 Operation of Defence/Pillar of Cloud conflict	(i) The integrated (whole-of-family) approach helped in reducing stress, increasing community engagement and provided more holistic support (ii) Inclusion of a psychosocial approach was fundamental in making the livelihoods intervention successful	
Eltayeb et al. (2017)	Sudan	Mental health and psychosocial intervention using IASC guidelines on mental health and psychosocial support in emergency settings (2007) and WHO 2001 mental health recommendations Cognitive Behaviour Therapy	Trauma related disorders such as PTSD and suffering as a result of exposure to war related violence and loss	International experts first trained mental health professionals who then became core trainers, going on to replicate training in other states	Acceptability Penetration	(i) Training in trauma case management, cognitive- behaviour competencies, referral and supervision (ii) Modules and training in lay counselling and psychological first aid (PFA) (iii) Modules and training in community healing using narrative theatre	Capacity building and narrative theatre activities Education sector	Trauma centre serving war affected Sudanese communities and building capacity of mental health professionals and psychosocial staff already working with displaced people	Individuals and communities, including internally displaced people affected by the civil war in Sudan	(i) Successfully trained 3 levels of personnel (specialised services, non-specialised services and community and family support) (ii) Tailored intensity according to the cohort delivering the intervention	(i) Staff turnover of trained personnel (ii) Follow up challenges with committees formed after each narrative theatre event (iii) Lack of resources to commute and follow up
James et al. (2020)	Haiti	*Manual focusing on reducing mental health symptoms*,* increasing functioning*,* increasing use of coping skills*,* reducing mental health stigma*,* increasing mental health knowledge*	Depression, PTSD, anxiety, functional impairment	2 trained Haitian lay mental health workers delivering a mental health integrated disaster preparedness intervention	Acceptability Penetration	Training of lay mental health workers	Disaster preparedness	Community-based disaster mental health intervention	Earthquake exposed and flood-prone communities as there are natural hazards in Haiti		
Weine et al. (2021)	Turkey	Low Intensity Family Support (LIFS) based on stress reduction as applied in the Problem Management Plus (PM+) intervention	Common mental disorders	Trained lay service providers who are Syrian refugees delivering an intervention in a multiple family group: 6–8 families meet together for 4 weekly 2 h group sessions	Acceptability Feasibility	Task sharing	Family resilience from the family support and education group model called Coffee and Families Education and Support (CAFES) Education sector	The 4 intervention sessions combined and adapted components from both PM + and CAFES	Syrian refugees currently residing in Turkey, having fled from war in Syria Family support and education	(i) Coalition between academic and humanitarian organisation partners built to strengthen relations (ii) Research capacity of local researchers and partners built (iii) Family Support Design Team (FSDT) started to support in adapting LIFS manual	(i) General challenges in conducting research in humanitarian emergency settings (ii) Challenges in finding a psychiatrist who would accept the task-sharing approach
Welton-Mitchelle et al. (2018)	Nepal	*Manual focusing on reducing mental health symptoms*,* increasing functioning*,* increasing use of coping skills*,* reducing mental health stigma*,* increasing mental health knowledge*	Depression and PTSD	Trained Nepalese clinicians delivering a 3 day intervention focused on enhancing disaster preparedness, mental health and community cohesion	Effectiveness	Training of facilitators over a period of 2 weeks Adaptation of intervention for Nepal	Disaster preparedness	Community based group intervention that was culturally adapted, incorporating coping skills and community building activities	Earthquake affected communities after the 2015 earthquake in Nepal		

### Analyses and quality appraisal

All studies were critically appraised for quality and rigor using the mixed methods appraisal tool (MMAT) (Additional file 5) [30]. The MMAT is used to describe the process of study selection, the number of studies identified, included and excluded, and the reasons for the exclusion of quantitative, qualitative and mixed research designs. It begins with two screening questions, which we decided against using as the selection criteria of our review is limited to empirical studies. We began with the second step, which includes five sections to be completed depending on the category of the study designs. Analysis involved a narrative synthesis of findings structured around the parameters for data extraction of the included studies, namely, population characteristics, intervention characteristics described, and outcomes studied, which were used to summarise the studies included.

## Results

### Search results

**Fig. 1:**
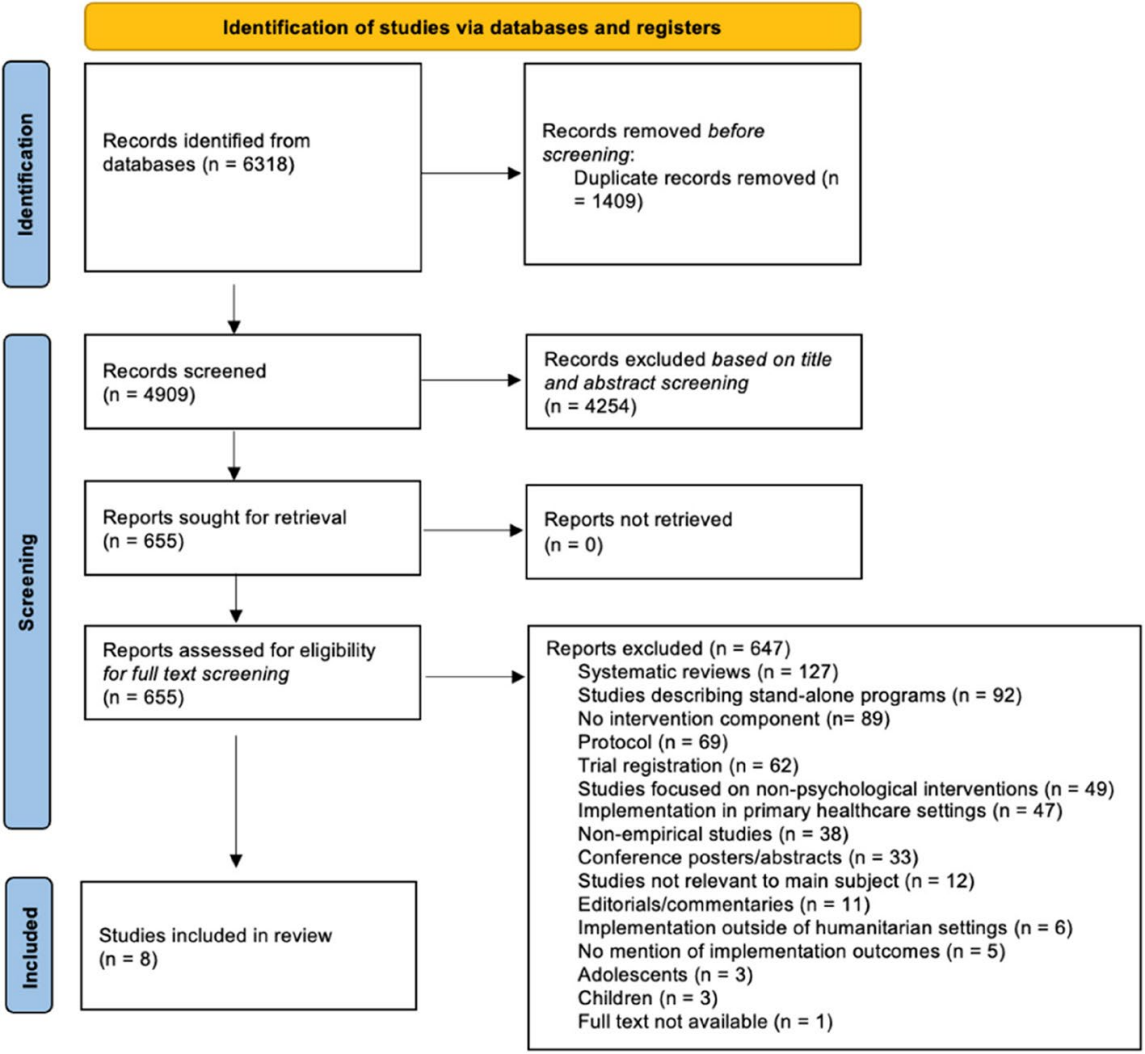
Flowchart of the retrieval, deduplication, exclusion, and inclusion of studies

A total of 6318 studies were identified through database searching. After deduplication, 4909 publications were screened, with eight meeting the eligibility criteria [31–38]. Details of the screening process are provided in Fig. 1. (PRISMA flow diagram).

### Study characteristics

Key characteristics of the studies are presented in Table 2. The eight studies included in this review were published between 2014 and 2022. Within the eight eligible studies, psychological interventions were integrated with protection services in Tanzania [36–38], livelihoods in the Gaza region [31], microfinance and income generating activities in Sudan, family support and education in Turkey [34], and disaster preparedness interventions in Nepal and Haiti [33, 35]. Seven of the eight studies were conducted in low and middle income countries (LMICs) [31, 32, 34–38]. Populations targeted included people experiencing, or who had experienced, a wide range of humanitarian crises, such as people living in active conflict areas [31], refugees who had settled in host countries after fleeing conflict [34, 36–38], internally displaced war-affected communities [32], and communities recovering from disasters such as those triggered by floods and earthquakes [33, 35]. The psychological interventions used in the studies were varied, ranging from cognitive processing therapy (CPT), problem management plus (PM+) to cognitive behaviour therapy. Some of the studies described in detail how and why adaptations of evidence based interventions such as CPT and PM + were made [34, 36–38]. Only three of the studies were explicit in detailing implementation outcomes and how they were ascertained [36–38], and based on descriptions, the other five studies might have used the Proctor checklist or similar to describe implementation processes. Acceptability was the only outcome described across all studies. While one study used a quantitative approach to ascertain a broad range of different implementation outcomes [38], three studies qualitatively described a selection of implementation outcomes, and four studies focused on a mixed-methods approach [33–35, 37].

### Types of psychological interventions

The primary focus across all studies was on the treatment of psychological distress and/or CMDs, and a few other studies also incorporated mental health promotion and prevention as illustrated in Table 3.

**Table 3:**
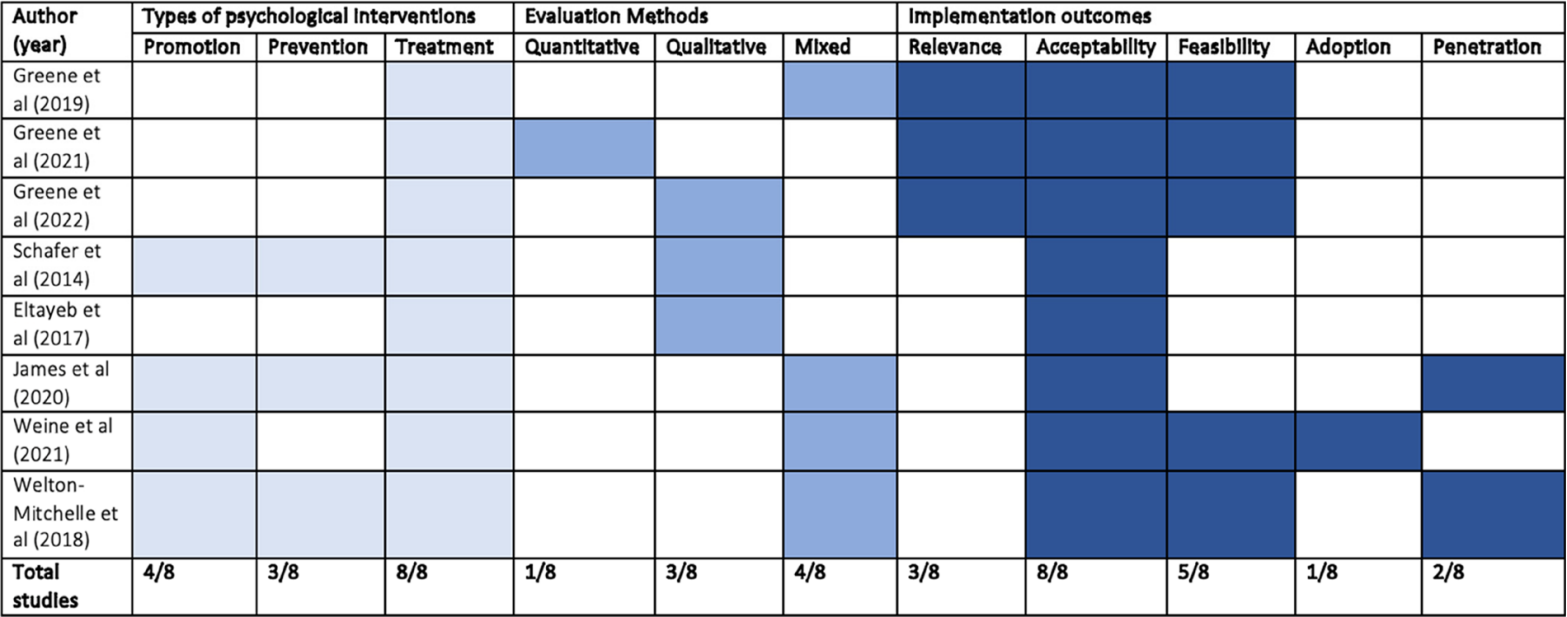
Summary of psychological intervention types, evaluation methods and implementation outcomes of included studies

One umbrella project, of which three of the included studies are products, focused on a mental health intervention for intimate partner violence (IPV) survivors that targeted psychological distress broadly [36–38]. An adapted version of cognitive processing therapy, a psychological intervention developed to treat mental health outcomes in survivors of sexual violence, was integrated with advocacy counselling, an intervention that has shown reductions in IPV in prior evaluation studies. Results indicated reductions in some indicators of psychological distress and IPV severity, but these were moderate compared to other studies where these interventions were implemented independently (CPT and advocacy counselling).

In another study, an intervention focused on the promotion, prevention and treatment of trauma related disorders, drawing on the IASC guidelines developed in 2007 and the WHO 2001 recommendations developed in the context of exposure to war-related violence and loss [32]. The intervention was delivered in three parts, which collectively help to better understand the intervention as a whole. The first part focused on training mental health professionals in understanding trauma and trauma counselling competencies using a cognitive behaviour therapy (CBT) approach. The second part included service provision, where trauma mental health centres were set up to provide specialised services to communities. The third part of this intervention integrated psychological interventions with narrative theatre to educate families and communities about mental health through drama activities.

Two articles focused on a hybrid mental health and disaster preparedness intervention for earthquake and flood survivors [33, 35]. One study, conducted in Nepal [33], based their adapted intervention on a model initially developed for earthquake survivors in Haiti with promotion, prevention and treatment characteristics [35]. Results across the studies showed an increase in disaster preparedness and also showed reduced symptoms associated with depression, PTSD, and anxiety with small to moderate effect sizes.

A novel transdiagnostic intervention for refugee families with common mental disorders, called low intensity family support (LIFS) merged components from problem management plus (PM+) and a family resilience approach, adapted from a family support and education intervention [34]. The study focused on challenges encountered in developing and piloting the LIFS intervention; thus, mental health outcomes were not measured. Results indicated challenges encountered in creating strong partnerships, implementing task sharing approaches, overcoming language and culture barriers, gender dynamics and working in a fragile context.

A World Vision livelihoods project integrated mental health into their programming through training agricultural extension officers in psychological first id (PFA), to support farmers while also offering practical assistance through their livelihoods work [31]. The intervention extended to mothers who had children attending World Vision child-friendly spaces, facilitators in their projects, farmers and youth mentors, and they were also trained in PFA, psychosocial awareness and other educational sessions on various topics. From this study, there were strong indications that there was an association between livelihoods and psychosocial support based on the outcomes. Benefits from the integrated intervention, such as less family stress and greater family harmony, were attributed to the whole-family approach used from children, mothers, fathers, and the people working with them and supporting them.

### Description of integration processes

Table 2 (key study characteristics) includes a summary that describes the integration process of the studies included. All studies employed some form of integration of evidence-based psychological intervention elements, which have previously been shown to reduce mental health challenges, with other elements from interventions widely used in other sectors, which have also been effective in their respective fields.

#### Conceptualisation of integrated interventions

As integration is a relatively new and untested pathway for intervention delivery, most studies (five) began the process by learning more about how best to combine different intervention elements. This was often achieved through reviewing literature to understand current practices, site visits to understand the context and engage with local stakeholders and formative qualitative research that entailed consultations with communities and experts [33, 34, 36–38]. Three studies reported establishing a community advisory board consisting of different stakeholders such as village elders, experts and camp commanders whom the research team could consult with throughout the project to guide the implementation approach [36–38]. Two of the nine studies integrated interventions using a culturally informed approach, and adaptations were done with the local communities, intervention facilitators and other stakeholders who understood the context [33, 35].

#### Design and adaptation of integrated interventions

Due to the multisectoral nature of the interventions, changes were made to enhance implementation at various time points, from development to evaluation. Three studies adapted individual intervention components, for example, shortening the original interventions to accommodate other elements that were being incorporated [36–38]. Two studies chose to implement the interventions that had already been integrated, and they chose to test the integrated interventions with different populations [33, 34]. In these cases, only local adaptations, such as language and other context-specific adaptations were made, and the integrity of the original integrated interventions was maintained. In three studies, adaptations that are considered good practice based on consensus-based recommendations, such as translation to local languages and adaptation of original intervention delivery to fit in with the local context, were made while developing the integrated intervention to increase the feasibility of the integrated intervention versions [36–38].

#### Implementation through task shifting

Service delivery was an aspect considered across all interventions. Those delivering the interventions were mostly lay workers in five of the eight studies, who had received brief training and were continuously supervised and supported during implementation. Three of the studies differed in this aspect, and instead used trained mental health professionals as core providers [32, 33, 35]. Mental health professionals were also trained first by international experts, and they went on to replicate the training they received in other states through a trainer of trainer’s model. Models of compensation for both those delivering the interventions and participants were seldom reported. When reported in two studies, participants received token compensation such as a radio [35], lunch and local travel provision [33], and one study also provided participants with disaster supply kits [33]. Three articles focusing on the same intervention reported on compensation for lay refugee incentive workers delivering the intervention, including fixed compensation in line with local laws on refugee employment, which was also identified as a barrier to implementation [36–38].

#### Partnerships and capacity development

In addition, both inter-sectoral and interdisciplinary partnerships also played a role in the integration process. Organisations and researchers that did not have the expertise and capacity in another field opted to partner with those working in other sectors, e.g., livelihoods, that could ensure that integrated interventions would be delivered at very high quality without compromise [31]. In some aspects, facilitators were trained by people with expertise in various disciplines to complement the integrated approaches of the interventions and to ensure that capacity was developed and strengthened. For example, a clinical psychologist and a medical anthropologist delivered facilitator training to local refugee incentive workers in one study [37]. The clinical psychologist had expertise in trauma-informed psychological interventions, relevant to supporting the mental health components of the integrated intervention. On the other hand, the medical anthropologist had expertise in community psychiatric nursing and gender-based violence, with competences to support both mental health and intimate partner violence components.

In one study focusing on specialised services, mental health professionals were trained in trauma and case management [32]. They also trained non-governmental organisation (NGO) workers and government officials working in non-specialised services such as NGOs, and others at the community and family support level were also trained. The rationale behind training these groups of people first, before implementing the integrated intervention in communities, was that people who had more severe mental health needs would need to be referred to services.

### Description of implementation indicators and outcomes

As summarised in Table 3, a total of five out of eight implementation outcomes, as described by Proctor and colleagues, were reported across the studies [28]. These are relevance, acceptability, feasibility, adoption, and penetration. It is apparent from this table that a key implementation outcome of importance reported across the studies concerned the acceptability of the integrated interventions; all studies investigated this outcome. In comparison, only four of the nine studies investigated other implementation outcomes (relevance and feasibility); one study examined adoption; and two of the studies investigated penetration.

#### Acceptability

Indicators applied to assess acceptability included participant retention rates and qualitative descriptions of the benefits and challenges of the intervention as perceived by participants, facilitators, implementers and other stakeholders [32–38]. Session content, delivery and length were also considered key in establishing whether integrated interventions were acceptable, including how they fit in the broader context, taking into account social, cultural, and organisational contexts [34].

#### Feasibility

Five of the eight studies linked task shifting and/or task sharing strategies as part of their strategy to enhance feasibility [31, 34, 36–38]. Although not mentioned explicitly, task shifting and/or task sharing were also linked to sustainability. One study trained mental health professionals to continue training other health professions across the country in an effort to build capacity on a larger scale. Service providers were generally lay refugee workers [34–38]. Facilitators also included people who traditionally do not interact with or participate in mental health interventions, such as agricultural extension officers [31]. Three studies differed in their selection of facilitators, and they opted to use mental health professionals and clinicians who had a mental health background that helped in understanding processes of training and implementation [32, 33, 35]. All service providers underwent some form of training across different levels of influence. Some of the studies chose to enhance feasibility by using integrated interventions that had previously been used in different settings where relevance to those populations was already established [33, 35].

#### Relevance

The relevance of integrated interventions in the different settings was also explored through mixed methods such as desk reviews and formative qualitative interviews with stakeholders. An indicator of relevance mentioned in one study was the burden of problems in target populations to establish need [38]. The study used psychometric evaluation of outcome measures e.g. Hopkins Symptoms Checklist, Abuse Assessment Screen and Harvard Trauma Questionnaire, to assess the target impact group’s distress and IPV [38]. With needs firmly established, some of the ways to improve the relevance of the integrated interventions included cultural adaptations to improve intervention acceptability to target impact groups based on consultations with local stakeholders with contextual and social knowledge [33, 36–38]. Local knowledge and actively including local participants in developing and adapting integrated interventions appeared to greatly improve implementation outcomes across the studies.

#### Adoption

Only one study investigated adoption as an implementation outcome [34]. One of the aims of the study focused primarily on how group facilitators and community-based organisations could best overcome obstacles to promote engagement and retention in the integrated intervention groups. Strategies to enhance adoption, and by extension, integration as mentioned, included initiating and developing collaborations and partnerships, both across and within sectors. The researchers looked for collaborators who had scientific and practical experiences in the context, which strengthened the research team and intervention uptake.

#### Penetration

A mental health integrated disaster preparedness intervention conducted in Haiti effectively improved mental health and preparedness among community members who were vulnerable to natural hazards [35]. Building on this study, another study was conducted in Nepal among earthquake-affected communities, which adapted the Haiti-model, exemplifying cross-border replication of an integrated intervention [33]. The study in Haiti essentially “spread” to Nepal, highlighting a level of penetration across borders and across target impact groups.

## Discussion

In this review, we sought to review current evidence for integrating psychological interventions with non-health sectors. Our review identified relatively few studies in this area. This may be an indication that, while multi-sectoral integration of psychological interventions with non-health sectors may be common in practice, little research is being done to rigorously document these processes and experiences.

Our findings point towards mixed results, with some aspects of multisectoral integration contributing to positive implementation outcomes, while others remain undetermined. There are indications that integrated multisectoral interventions may be an acceptable and feasible means of increasing reach in humanitarian crisis settings [1, 9]. Our results indicated that one key facilitator of successfully integrating interventions included ensuring that implementing staff were adequately trained and had access to support through supervision throughout implementation, as task-shifting approaches were mostly used. Other key facilitators included building strong interdisciplinary and inter-sectoral partnerships and collaborations, investing in capacity development, and ensuring that intervention elements align with local needs. These findings are consistent with studies on integrated interventions drawing on components from health, including HIV and cancer, and intimate partner violence [26, 39–41]. There have been examples where similar activities have been conducted in maternal and child nutrition where multisectoral integration and approaches have been incorporated within policies, guidelines, implementing organisations and community practice to guide successful implementation for healthy growth in children [15, 42, 43]. The humanitarian sector and mental health field could learn from such examples to build frameworks that anchor best practices that can guide how we not only approach multisectoral integration, but also how we can instil practices into systems that can be adopted and adapted globally.

Overall, calls for multisectoral integration in mental health have been growing stronger since the world health report in 2001 recognised mental health as under resourced and neglected within health [44]. The report stressed that good mental health was crucial for the overall health and wellbeing of people, from individual level to societal level, and had to be universally regarded as such. The primary focus in the humanitarian research literature has been on integrating psychological interventions into the primary health care system. Although integrating psychological interventions within health is important, our findings show that we also need to integrate psychological interventions with interventions outside of the non-health sector. It is important to ensure that mental health is a priority in sectors beyond the health system. Moreover, actors working in mental health also have to recognise the role other sectors play in mental health and incorporate integrated perspectives into their approaches [21, 45]. Additionally, there is growing recognition that mental and social needs in humanitarian settings may be strongly intertwined in bi-directional ways [46].

Integrated multisectoral interventions are often adapted based on the context in which they are being implemented. However, without evaluation, the impacts of modifying interventions remain unknown. As separate stand-alone interventions, the evidence base on the effectiveness of these intervention components is typically well established. However, the impacts on treatment or prevention outcomes of delivering multisectoral, integrated interventions that have been modified to increase feasibility, for example, are less known. Results from this review have shown improvements in the primary outcomes investigated, but for most of the studies, effect sizes were smaller compared to previous interventions where efficacy was established [36–38]. The overall positive change in effect on mental health outcomes and other non-health outcomes at the end of an intervention might be due to complementary interactions when an intervention had been developed to address multiple concerns at the same time, such as in the positive outcomes identified in relation to integrated HIV and mental health interventions [47]. This is also consistent with findings from other types of multisectoral integrated interventions in poverty and food insecurity studies [48, 49], where reductions in both poverty and food insecurity were observed. Future research, where randomised control trial (RCT) designs are used to evaluate integrated multi-sectoral interventions should be considered to examine multi-sectoral impacts and examine mechanisms of impact.

One implementation aspect that was not explicitly mentioned across all the studies is sustainability and the approaches used to enhance it. Although not referred to specifically under this implementation outcome, it could be argued that task shifting could be perceived as a key strategy to enhance uptake and more sustained ways of delivering interventions in communities affected by conflict [50]. Although there are a number of positive aspects of task-shifting, such as expanding the capacity to address health worker shortages [51], and optimise use of existing health workers [33, 35], there are drawbacks to this approach that apply, particularly in humanitarian settings where mobility is high. One of the challenges faced in task shifting that affect sustainability includes difficulties in ensuring that the quality of intervention delivery is maintained and sustained over time. Others include resistance to task shifting and difficulties keeping staff motivated. Other challenges, which specifically apply to humanitarian settings, is high staff turnover and high mobility of trained facilitators. These findings are consistent with other interventions that have used task-shifting approaches, particularly in humanitarian settings [52].

It would be beneficial to incorporate sustainability into integrated interventions from the onset and limit some of the challenges faced. Multisectoral, integrated interventions require successful partnerships within and across sectors for them to be delivered successfully. However, as is often seen in interdisciplinary work, developing and sustaining partnerships is a challenge. In the example by Weine and colleagues, they faced challenges in finding programme partners with research capacity, and they also found that the academic partners had no relationship with humanitarian organisations. This divide, which is consistent with other findings between research and practice, reinforces how working in silos has historically not been beneficial. It also shows that for researchers and organisations that would want to break this chain and venture into multisectoral approaches, there is a need to understand the formative work that needs to be done to first to overcome barriers to multi-sectoral work. The multisectoral nature of the studies in this review itself poses challenges. Research on multi-sectoral integration requires bridging sector- and discipline-specific terminology, approaches, frameworks, and methodologies. Taking this into account, we started the review by conceptualising key terms and definitions. Although we found this exercise helpful in establishing common ground, it also highlighted a gap in multisectoral approaches that has the potential to diminish efforts that are being made to integrate different services from various sectors.

### Challenges faced and implications for future research

We have identified four key challenges that should be considered by future researchers in this field. First, high migration rates are to be expected, particularly when emergencies are ongoing. This mobility may affect staff turnover and lower intervention retention rates. Secondly, interdisciplinary and inter-sectoral work poses challenges in communication and coordination, affecting overall teamwork. Thirdly, task-shifting approaches, although helpful, are not widely accepted or universally appropriate. In humanitarian crisis settings, health staff and community workers may be overwhelmed and have conflicting responsibilities, particularly in situations where they may not be adequately remunerated. Fourth and lastly, social, cultural, and environmental contexts must be understood, respected, and adhered to. In some cases, this may include engaging stakeholders in the language they are comfortable with, organising interventions to meet age- and gender-specific needs, and ensuring family values are respected, among other considerations.

### Strengths and limitations

This review had several strengths and limitations. First, to our knowledge, this is the first review to synthesise literature on the integration of mental health interventions into non-health sectors in humanitarian settings. We also reached out to authors, inquiring about possible follow up studies that we could include in our review, ensuring that all possible studies were included. In addition to these strengths, there are some key limitations that should be considered. For this review, our search strategy was a modification and expansion of the one used by Troup, Fuhr, Woodward, Sondorp and Roberts. Although their work was insightful in shaping the review focus, we cannot exclude the possibility that we might have limited our scope in this manner as their review focused more on scaling up MHPSS interventions in low- and middle-income countries. Our review identified only eight studies and did not explore the grey literature. Another limitation is that we made minor changes from the preregistration of this systematic review, such as changes to the type of data extracted, e.g., adding key facilitators and challenges reported in studies, as this allowed for more comprehensive reporting on the studies. Some studies included in our review were not explicit in reporting implementation outcomes and strategies, which limited our assessment of these factors to an analysis of implicit language and general descriptions consistent with implementation science definitions.

## Conclusion

The present review is the first to summarise how psychological interventions in humanitarian crises settings globally have been implemented through integration into non-health sector programming for people at risk of, and/or experiencing, psychological distress and CMDs. All studies explored acceptability while five studies focused on feasibility of the integrated interventions. Other implementation outcomes were explored to a lesser extent. A small number of studies identified in this area demonstrated improvements in mental health treatment, prevention, and promotion outcomes and associated non-health outcomes were observed; however, mechanisms of change have remained largely undetermined. As an initial step into exploring integrated multisectoral interventions as a complimentary means of implementation and scaling of psychological interventions, we can learn from the implementation strategies used and the different challenges faced to build blueprints for future integrative work across sectors in humanitarian settings.

## Supplementary Information

Supplementary Material 1.

## Data Availability

All data generated or analysed during this study are included in this published manuscript (and its additional files 1, 2, 3 and 4).

## Abbreviations

CBT: Cognitive Behaviour Therapy
CMDs: Common Mental Disorders
CPT: Cognitive Processing Therapy
IASC: Inter Agency Standing Committee
IPV: Intimate Partner Violence
LIFS: Low Intensity Family Support
LMIC: Low–and Middle–Income Country
MHPSS: Mental Health and Psychosocial Support
MMAT: Mixed Methods Appraisal Tool
PFA: Psychological First Aid
PM+: Problem Management Plus
PRISMA: Preferred Reporting Items for Systematic Reviews and Meta–Analyses
PTSD: Post Traumatic Stress Disorder
