# Supplementary material for: Integration of psychological interventions in multi-sectoral humanitarian programmes: a systematic review
Source: BMC Health Serv Res. 2024 Dec 2;24:1528. doi: 10.1186/s12913-024-11704-7 (PMC11613475; doi:10.1186/s12913-024-11704-7)
Supplement: Supplementary file 1 — Supplementary Material 1. [file 12913_2024_11704_MOESM1_ESM.docx]

### Supplementary files

### Additional file 1: PRISMA checklist

| **Section and Topic** | **Item #** | **Checklist item** | **Location where item is reported**  **Page #** |
| --- | --- | --- | --- |
| **TITLE** | | |  |
| Title | 1 | Identify the report as a systematic review. | 1 |
| **ABSTRACT** | | |  |
| Abstract | 2 | See the PRISMA 2020 for Abstracts checklist. | 2 |
| **INTRODUCTION** | | |  |
| Rationale | 3 | Describe the rationale for the review in the context of existing knowledge. | 3-5 |
| Objectives | 4 | Provide an explicit statement of the objective(s) or question(s) the review addresses. | 5 |
| **METHODS** | | |  |
| Eligibility criteria | 5 | Specify the inclusion and exclusion criteria for the review and how studies were grouped for the syntheses. | 5-7 |
| Information sources | 6 | Specify all databases, registers, websites, organisations, reference lists and other sources searched or consulted to identify studies. Specify the date when each source was last searched or consulted. | 6-7 |
| Search strategy | 7 | Present the full search strategies for all databases, registers and websites, including any filters and limits used. |  |
| Selection process | 8 | Specify the methods used to decide whether a study met the inclusion criteria of the review, including how many reviewers screened each record and each report retrieved, whether they worked independently, and if applicable, details of automation tools used in the process. | 7 |
| Data collection process | 9 | Specify the methods used to collect data from reports, including how many reviewers collected data from each report, whether they worked independently, any processes for obtaining or confirming data from study investigators, and if applicable, details of automation tools used in the process. | 7 |
| Data items | 10a | List and define all outcomes for which data were sought. Specify whether all results that were compatible with each outcome domain in each study were sought (e.g. for all measures, time points, analyses), and if not, the methods used to decide which results to collect. | 5-6 |
|  | 10b | List and define all other variables for which data were sought (e.g. participant and intervention characteristics, funding sources). Describe any assumptions made about any missing or unclear information. | 5-6 |
| Study risk of bias assessment | 11 | Specify the methods used to assess risk of bias in the included studies, including details of the tool(s) used, how many reviewers assessed each study and whether they worked independently, and if applicable, details of automation tools used in the process. | 7 |
| Effect measures | 12 | Specify for each outcome the effect measure(s) (e.g. risk ratio, mean difference) used in the synthesis or presentation of results. | N/A |
| Synthesis methods | 13a | Describe the processes used to decide which studies were eligible for each synthesis (e.g. tabulating the study intervention characteristics and comparing against the planned groups for each synthesis (item #5)). |  |
|  | 13b | Describe any methods required to prepare the data for presentation or synthesis, such as handling of missing summary statistics, or data conversions. | N/A |
|  | 13c | Describe any methods used to tabulate or visually display results of individual studies and syntheses. | 8 |
|  | 13d | Describe any methods used to synthesize results and provide a rationale for the choice(s). If meta-analysis was performed, describe the model(s), method(s) to identify the presence and extent of statistical heterogeneity, and software package(s) used. | N/A |
|  | 13e | Describe any methods used to explore possible causes of heterogeneity among study results (e.g. subgroup analysis, meta-regression). | N/A |
|  | 13f | Describe any sensitivity analyses conducted to assess robustness of the synthesized results. | N/A |
| Reporting bias assessment | 14 | Describe any methods used to assess risk of bias due to missing results in a synthesis (arising from reporting biases). | N/A |
| Certainty assessment | 15 | Describe any methods used to assess certainty (or confidence) in the body of evidence for an outcome. | N/A |
| **RESULTS** | | |  |
| Study selection | 16a | Describe the results of the search and selection process, from the number of records identified in the search to the number of studies included in the review, ideally using a flow diagram. | 8 |
|  | 16b | Cite studies that might appear to meet the inclusion criteria, but which were excluded, and explain why they were excluded. | 8 |
| Study characteristics | 17 | Cite each included study and present its characteristics. | 9 |
| Risk of bias in studies | 18 | Present assessments of risk of bias for each included study. |  |
| Results of individual studies | 19 | For all outcomes, present, for each study: (a) summary statistics for each group (where appropriate) and (b) an effect estimate and its precision (e.g. confidence/credible interval), ideally using structured tables or plots. | N/A |
| Results of syntheses | 20a | For each synthesis, briefly summarise the characteristics and risk of bias among contributing studies. | N/A |
|  | 20b | Present results of all statistical syntheses conducted. If meta-analysis was done, present for each the summary estimate and its precision (e.g. confidence/credible interval) and measures of statistical heterogeneity. If comparing groups, describe the direction of the effect. | N/A |
|  | 20c | Present results of all investigations of possible causes of heterogeneity among study results. | 9-14 |
|  | 20d | Present results of all sensitivity analyses conducted to assess the robustness of the synthesized results. | N/A |
| Reporting biases | 21 | Present assessments of risk of bias due to missing results (arising from reporting biases) for each synthesis assessed. | N/A |
| Certainty of evidence | 22 | Present assessments of certainty (or confidence) in the body of evidence for each outcome assessed. | N/A |
| **DISCUSSION** | | |  |
| Discussion | 23a | Provide a general interpretation of the results in the context of other evidence. | 15-16 |
|  | 23b | Discuss any limitations of the evidence included in the review. | 14-15 |
|  | 23c | Discuss any limitations of the review processes used. | 14-15 |
|  | 23d | Discuss implications of the results for practice, policy, and future research. | 17 |
| **OTHER INFORMATION** | | |  |
| Registration and protocol | 24a | Provide registration information for the review, including register name and registration number, or state that the review was not registered. | 5; 24 |
|  | 24b | Indicate where the review protocol can be accessed, or state that a protocol was not prepared. | 5; 24 |
|  | 24c | Describe and explain any amendments to information provided at registration or in the protocol. | N/A |
| Support | 25 | Describe sources of financial or non-financial support for the review, and the role of the funders or sponsors in the review. | 24 |
| Competing interests | 26 | Declare any competing interests of review authors. | 24 |
| Availability of data, code and other materials | 27 | Report which of the following are publicly available and where they can be found: template data collection forms; data extracted from included studies; data used for all analyses; analytic code; any other materials used in the review. | 25 |

### Additional file 2: Inclusion and exclusion criteria table

| Selection Criteria | Inclusion Criteria | Exclusion Criteria |
| --- | --- | --- |
| Study type | Quantitative, qualitative and mixed-methods (inclusive) | Systematic reviews (separately identified for reference list searching)  Editorials  Commentaries |
| Publication type | Peer-reviewed journals  PhD dissertations | Conference posters/abstracts |
| Populations | Adults above 18 years | Children aged below 18 years (with no overlaps in age)  Animal studies |
| Type of intervention | Studies that deliver intervention through multisectoral integration | Studies describing integration into healthcare settings or as stand-alone programs |
| Outcomes | At least one implementation outcome relating to improving acceptability, adoption, appropriateness, feasibility, fidelity, penetration and sustainability | No mention of implementation outcomes |
| Source of data | Studies reporting on original data | Non-empirical studies |
| Focus area | Studies focused solely on psychological interventions | Studies focused on non-psychological interventions |
| Publication date | All | None |

### Additional file 3: Examples of studies excluded

| **Exclusion criteria** | **Examples** |
| --- | --- |
| Studies describing stand-alone programs | [Singh et al, 2021](https://conflictandhealth.biomedcentral.com/articles/10.1186/s13031-021-00391-4); [Koch et al., 2020](https://regroup-production.s3.amazonaws.com/documents/ReviewReference/468018073/1-s2.0-S0005796720301431-main.pdf?response-content-type=application%2Fpdf&X-Amz-Algorithm=AWS4-HMAC-SHA256&X-Amz-Credential=AKIAYSFKCAWYQ4D5IUHG%2F20240523%2Fus-east-1%2Fs3%2Faws4_request&X-Amz-Date=20240523T110947Z&X-Amz-Expires=604800&X-Amz-SignedHeaders=host&X-Amz-Signature=af1dccfa8170f3cb59a3bca1038ef2d3030c107d98522ec8e030fd7ad24cfe5a); [Jordans et al., 2021](https://journals.plos.org/plosmedicine/article?id=10.1371/journal.pmed.1003621); [Rycroft-Malone et al., 2017](https://www.journalslibrary.nihr.ac.uk/hsdr/hsdr05140#/abstract); [Raviola et al., 2012](https://journals.lww.com/hrpjournal/abstract/2012/02080/mental_health_response_in_haiti_in_the_aftermath.9.aspx) |
| No intervention component | [Liebling et al, 2020](https://www.mdpi.com/1660-4601/17/5/1685); [Crepet et al, 2017](https://conflictandhealth.biomedcentral.com/articles/10.1186/s13031-017-0103-3); [Walther et al., 2021](https://bmcpublichealth.biomedcentral.com/articles/10.1186/s12889-021-10817-6); [Saka et al., 2017](https://journals.sagepub.com/doi/10.1177/0030222817732466); [Kagoyire & Richters, 2018](https://tidsskrift.dk/torture-journal/article/view/111183) |
| Studies forcused on non psychological interventions | [Story et al, 2020](https://www.sciencedirect.com/science/article/abs/pii/S0277953618304519?via%3Dihub); [Shah et al., 2017](https://www.sciencedirect.com/science/article/abs/pii/S0095454317300295?via%3Dihub); [Rasmussen et al., 2011](https://journals.sagepub.com/doi/10.1177/1363461511409283); [Moya et al., 2021](https://regroup-production.s3.amazonaws.com/documents/ReviewReference/468018421/Moya-2021-The%20COVID-19%20pandemic%20and%20maternal%20m.pdf?response-content-type=application%2Fpdf&X-Amz-Algorithm=AWS4-HMAC-SHA256&X-Amz-Credential=AKIAYSFKCAWYQ4D5IUHG%2F20240523%2Fus-east-1%2Fs3%2Faws4_request&X-Amz-Date=20240523T112852Z&X-Amz-Expires=604800&X-Amz-SignedHeaders=host&X-Amz-Signature=1405db54128b610a7f07b88104fb236b3f64896a5955a3afabfc21825a5886e6); [Pizzi et al., 2014](https://agsjournals.onlinelibrary.wiley.com/doi/10.1111/jgs.13146) |
| Implementation in primary healthcare settings | [Salize et al, 2014](https://www.frontiersin.org/journals/public-health/articles/10.3389/fpubh.2014.00026/full); [Trilesnik et al., 2019](https://www.frontiersin.org/journals/psychiatry/articles/10.3389/fpsyt.2019.00688/full); [Hansen, 2015](https://bmccomplementmedtherapies.biomedcentral.com/articles/10.1186/s12906-015-0613-8); [Sajatovic et al., 2019](https://onlinelibrary.wiley.com/doi/10.1111/epi.16322); [Cianelli et al., 2013](https://onlinelibrary.wiley.com/doi/10.1111/inr.12047) |

### Additional file 4: Search strategy

We grouped the search terms in four main clusters consisting of subclusters that we combined with Boolean operators:

• populations (people at risk of/or experiencing psychological distress and common mental disorders (CMDs) (i.e., depression, anxiety, PTSD, and somatoform conditions) – sets 1-10

• interest (psychological interventions, integration, non-health sectors) – sets 11-16

• context (humanitarian settings globally) – sets 17-22

• methodology i.e., qualitative, quantitative or mixed studies – sets 23-25

In other search systems than Ovid Medline ALL, relevant adaptations were made, for instance the use of system specific subject headings. Also, this search strategy was adapted to different search systems in other ways:

- Ovid Evidence Based Medicine Reviews: not subject headings were used
- PTSDpubs: the search strategy is too complicated for this search system (in ProQuest), so I simplified it considerably by using mainly subject headings.
- a number of search terms referring to trauma without any qualification as to whether they are mental or physical, are only used in PsycINFO and not in Ovid Medline, Embase and EBMR: (Trauma or multitrauma or traumatised or traumatized or (related adj3 Trauma*).mp. . The terms (related adj3 Trauma*).mp. are used in Embase and EBMR, however.

Key to abbreviations used:

Ovid: "/"=subject heading, exp = explode: search for the subject heading and also its narrower, more specific terms, mp=multi purpose field, comprises title, abstract, original title, heading terms or key concepts, table of content, test and measures.

PTSDpubs: MAINSUBJECT.EXACT.EXPLODE = subject heading, exploded, ti=title, ab=abstract, su=subject heading.

#### Ovid Medline ALL

16/02/2022

**Ovid MEDLINE(R) ALL <1946 to February 15, 2022>**

| Search history sorted by search number ascending | | | |  |  |  |
| --- | --- | --- | --- | --- | --- | --- |
| **#** | **Searches** | **Results** | **Type** |  |  |  |
|  | | | | | | |
| 1 | exp Psychological Distress/ or exp "Stress, Psychological"/ or exp "Adaptation, Psychological"/ or exp "Resilience, Psychological"/ or ("common mental disorder*" or CMD or well-being or wellbeing or wellness or burnout or coping or cope or endurance or Hardiness or Resilien* or ((psych* or emotion* or mental or chronic or life or behavio* or social* or interpersonal* or nervous*) adj3 (stress* or distress* or exhaust* or pressure* or shock* or tension*)) or ((dystress or distress) adj3 syndrome*) or (burn adj3 out) or ((occupational or job or work* or professional* or career* or school) adj3 (stress* or distress* or bully* or abuse*)) or ((financ* or Economic*) adj3 (stress* or burden* or hardship* or challenge* or pressure* or toxicit* or strain* or distress* or crisis or crises)) or ((caregiver* or care) adj3 (burden* or exhaust* or strain*)) or ((family or families or familial or home or parent* or maternal* or paternal* or mother* or father*) adj3 (stress* or distress* or crisis or crises or strain*)) or ((moral* or ethic*) adj3 (stress* or distress* or dilemma* or doubt* or paradox* or injur*)) or (critical-incident* adj3 (stress* or distress*)) or ((adapt* or adjust*) adj3 (behavio* or emotion* or psych*))).mp. | 748614 | Advanced |  |  |  |
| 2 | mental fatigue/ or (((mental* or emotional* or psych* or cogniti* or stress*) adj5 (Fatigue* or exhaust* or Lassitud*)) or (low adj3 alert*)).mp. | 18195 | Advanced |  |  |  |
| 3 | exp Anxiety/ or (Anxiet* or Angst or Hypervigilan* or Nervous* or Anxious* or Catastrophi* or fear or fearing or fears).mp. | 922634 | Advanced |  |  |  |
| 4 | Sadness/ or (Sadness or Unhappiness or sad or unhappy or melanchol*).mp. | 21946 | Advanced |  |  |  |
| 5 | (social adj2 (avoid* or anxiet* or anxious*)).mp. | 10983 | Advanced |  |  |  |
| 6 | anxiety disorders/ or "anxiety, separation"/ or neurocirculatory asthenia/ or neurotic disorders/ or panic disorder/ or (GAD or ((anxiet* or anxious*) adj3 (disorder* or generalized or generalised)) or (Anxiety adj1 Separation) or cardioneuros* or effort-syndrome or neurosis-cordis or ((cardiac or heart) adj2 (anxiet* or neuros*)) or (heart adj1 (soldier* or complaint or neurogenic)) or (neurocirculat* adj1 (astheni* or dystoni*)) or neuroses or neurosis or neurotic* or Psychoneuro* or panic* or cothymi* or ((anxiety or anxious) adj2 depressi*) or psychastheni* or (asthenic adj1 syndrome) or koro).mp. | 191287 | Advanced |  |  |  |
| 7 | exp "Stress Disorders, Traumatic"/ or exp "posttraumatic growth, psychological"/ or (psychotrauma* or PTSD or DES*NOS or C*PTSD or EPCACE or DTD or "Enduring Personality Change after Catastrophic Experience*" or (Stress adj3 disorder*) or ((combat or war) adj3 (experience* or disorder* or fatigue or neurosis or neuroses or stress)) or ((Emotional or Complex or chronic or Complicated or Multiple or related) adj3 Trauma*) or ((acute or military) adj3 Stress) or ((Stress or Crisis) adj3 Reaction*) or ((Post-Traumatic or posttraumatic or Trauma*) adj3 (stress or neurosis or neuroses or syndrome* or Disorder* or psychosis or psychoses or distress* or growth)) or (moral adj2 injur*)).mp. | 131051 | Advanced |  |  |  |
| 8 | exp Depressive Disorder/ or exp Depression/ or (depression* or depressive or depressed or dysthymia or dysthymic or mdd).mp. | 565467 | Advanced |  |  |  |
| 9 | exp "Bipolar and Related Disorders"/ or (maniodepressi* or cyclothymi* or (bipolar adj3 (psychos* or depress* or disorder*)) or (depressi* adj3 (mania or manic or mano))).mp. | 58885 | Advanced |  |  |  |
| 10 | 1 or 2 or 3 or 4 or 5 or 6 or 7 or 8 or 9 | 2088239 | Advanced |  |  |  |
| 11 | exp Mental Health Services/ or exp psychotherapy/ or mental health recovery/ or exp "Hospitals, Psychiatric"/ or (Psychotherap* or rehabilitat* or Hypnotherap* or Logotherap* or Psychoanaly* or Psychodrama* or Self-Analy* or Mindful* or CBT or CGT or EMDR or NET or KIDNET or BEPP or psychiatrist* or ((Group or psycho or Hypno or Logo or Mental or ACT or acceptance or aversion or Desensitization or Family or sex or management or Play or Primal or Schema or Couples or Gestalt or Insight or Network or Reality or Bowenian or Conjoint or Exposure or Ego or Feminist or Cognitive or cognition or Narrative or Strategic or Conversion or Persuasion or Affirmative or Existential or Relationship or Multisystemic or Client or Focused or Relational or Behavior* or Behaviour* or Constructivist or Centered or Rational or Emotive or Imagery or Rehearsal or Processing or Reciprocal or Inhibition or Interoceptive or Multiple or Systematic or Trauma*) adj3 Therap*) or ((psycho or self or Dream or Transactional) adj3 analy*) or (psycho adj3 drama) or (Age adj3 Regression) or (Chair adj3 Technique) or (Family adj3 Counseling) or (Guided adj3 Imagery) or (Autogenic adj3 Training) or (Methadone adj3 Maintenance) or (Therapeutic adj3 Community) or (Posthypnotic adj3 Suggestion*) or (Cognitive adj3 Restructuring) or (Contingency adj3 Management) or (Guided adj3 Imagery) or (Motivational adj3 Interviewing) or (Neurolinguistic adj3 Programming) or (Seeking adj3 Safety) or (Self adj3 Psychology) or (Stress adj3 Treatment) or (Traumatic adj3 Reduction) or (Virtual adj3 Exposure) or ((mental or family or trauma*) adj3 (service* or program* or intervention* or care or healthcare or support or healing)) or "safe space" or ((brief or low-intensity) and (CBT or intervention* or therap*)) or (psychiatric adj3 (hospital* or service*))).mp. | 1183381 | Advanced |  |  |  |
| 12 | exp Social Work/ or exp Social Welfare/ or exp Self-Help Groups/ or Social Support/ or Empowerment/ or Self Care/ or exp animal assisted therapy/ or exp art therapy/ or exp bibliotherapy/ or dance therapy/ or music therapy/ or exp occupational therapy/ or exp recreation therapy/ or exp "rehabilitation, vocational"/ or Psychiatric Rehabilitation/ or Crisis Intervention/ or Psychosocial Intervention/ or exp Counseling/ or exp virtual reality/ or (psycho-social or Psychosocial or MHPSS or ((intervention* or therap* or support or rehabilit*) adj5 (mental* or psych* or social* or animal or art or dance or music or occupational or recreation*)) or bibliotherap* or psychologist* or Psycho-education* or psychoeducation or PFA or communities or community* or empower* or counsel* or casework* or (Mental adj3 (center* or centre* or program*)) or (social adj3 (work* or psycholog* or service* or therap*)) or (psycholog* adj3 aid)).mp. | 1505931 | Advanced |  |  |  |
| 13 | ((prevent* or prophyla* or ((primary or early) adj5 (intervention* or care or service* or treatment))) and (mental* or psych* or social*)).mp. | 389662 | Advanced |  |  |  |
| 14 | 11 or 12 or 13 | 2525894 | Advanced |  |  |  |
| 15 | capacity building/ or (implement* or integrat* or ((scale or scaling or scales) adj1 (up or out or deep)) or (capacit* adj3 build*) or (task* adj3 (share* or sharing))).mp. | 1188912 | Advanced |  |  |  |
| 16 | exp Relief Work/ or Refugee Camps/ or Emergency Shelter/ or exp Food Security/ or exp Food Insecurity/ or exp Food Assistance/ or Water Insecurity/ or exp Water Supply/ or exp Water Quality/ or exp Sanitation/ or exp Hygiene/ or exp Education/ or exp Housing/ or exp Social Planning/ or exp Social Environment/ or ((water and sanitation and hygiene) or ((WIC or WASH or SNAP) adj2 program*) or ((food or water) adj3 (program* or aid or assistance or insecurit* or securit* or quality or inspect* or ration* or stamp* or healthy or suppl*)) or (nutrion* adj3 (program* or intervention* or assistance)) or protection or (protective adj3 (service* or program*)) or shelter* or ((water or wastewater) adj3 (Decolori* or Purification or softening or treatment)) or ((water or Artesian) adj1 well*) or sanitation or sanitary or latrine* or lavator* or restroom* or toilet* or plumbing or Hygiene or Hygienic or Disinfect* or ((waste or garbage or refuse) adj3 (disposal* or dump* or site* or ground* or manage* or Incinerat*)) or landfill* or education* or training or workshop* or college* or school* or teacher* or teaching or university or universities or (literacy adj1 program*) or ((refugee* or coordination or management) adj1 camp*) or housing or Lodging* or (communit* adj3 (care or network* or develop* or support*)) or ((social or psychosocial or neighbour* or neighbor*) adj3 (care or context* or ecolog* or environment* or integrat* or planning or support*)) or ((city or cities or town* or urban or rural or neighbourhood* or neighborhood*) adj3 (development or plan* or renewal*)) or (development adj3 plan*) or ((relief or aid or rescue) adj2 work*) or (humanitarian adj2 (relief* or response* or agenc* or aid))).mp. | 3217354 | Advanced |  |  |  |
| 17 | (humanitarian adj2 (crisis or crises or setting* or emergenc* or area*)).mp. | 1363 | Advanced |  |  |  |
| 18 | exp disasters/ or exp Starvation/ or (disaster* or "critical incident*" or avalanche* or earthquake* or groundshaking* or "mass movement" or Liquefaction* or Volcanic or volcano* or "ash fall" or lahar* or ((pyroclastic or lava) adj1 flow*) or flood* or landslide* or tsunami* or ((tidal or action* or rogue) adj1 wave) or seiche* or typhoon* or cyclone* or hurricane* or storm* or surge or surges or tornado* or wind* or rain* or blizzard* or derecho* or lightening* or thunderstorm* or hail* or sand or sandstorm* or duststorm* or drought* or "extreme temperature*" or "heat wave*" or heatwave* or "cold wave*" or coldwave* or "severe winter condition*" or snow* or ice* or frost* or freeze* or dzud or drought* or fire* or wildfire* or "wild fire*" or landfire* or "land fire*" or (fire* and (brush* or bush* or pasture* or forest*)) or bushfire* or "forest fire*" or (glacial and outburst*) or starvation or famine* or ((natural or Geological or hydrological or meteorological or climatological or Biological or Extraterrestrial or Human-induced or man-made or Technological or Societal) adj1 (accident or accidents or hazard* or crisis or crises or emergenc*)) or "insect infestation*" or grasshopper* or locust* or "foodborne" or "food borne" or (Extraterrestrial and impact) or airburst* or "space weather" or "energetic particles" or "geomagnetic storm*" or shockwave* or "Industrial hazard*" or "chemical spill*" or "gas leak*" or collapse* or explosion* or (industrial and fire*) or "nuclear accident*" or radiation or ((structural or building* or dam or dams or bridge*) adj1 (collapse* or failure*)) or ((transportation or air or road or rail or water) adj1 (accident* or crash*)) or "Air pollution*" or haze or "Power outage*" or "hazardous material*" or ((hazard* or pollution*) adj1 (biological or chemical or radiological)) or "food contamination*" or "financial crisis" or hyperinflation or "currency crisis" or massacre or bomb* or evacuation).mp. | 1521327 | Advanced |  |  |  |
| 19 | exp "Warfare and Armed Conflicts"/ or (war or wars or warring or warfar* or ((armed or zone or area*) adj2 conflict) or ("conflict affected" adj3 (population* or person* or communit*)) or (post adj2 (conflict* or emergenc*)) or "civil unrest" or terrorism or cbrn or cbrne or "chemical, biological, radiological, nuclear and explosive weapons" or "chemical, biological, radiological and nuclear").mp. | 125289 | Advanced |  |  |  |
| 20 | exp Disaster Victims/ or (disaster* adj3 (victim* or affected or survivor*)).mp. | 2131 | Advanced |  |  |  |
| 21 | refugees/ or (refugee* or asylumseek* or IDP* or (asylum adj3 (seek* or political)) or ((forced or irregular) adj3 (migrat* or migrant*)) or (displaced adj3 person*)).mp. | 20676 | Advanced |  |  |  |
| 22 | 17 or 18 or 19 or 20 or 21 | 1645339 | Advanced |  |  |  |
| 23 | ("Brain Imaging" or "Clinical Case Study" or "Clinical Trial" or "Empirical Study" or "Experimental Replication" or "Field Study" or "Focus Group" or "Followup Study" or "Interview" or "Longitudinal Study" or "Mathematical Model" or "Nonclinical Case Study" or "Prospective Study" or "Qualitative Study" or "Quantitative Study" or "Retrospective Study" or "Scientific Simulation" or "Treatment Outcome" or "Twin Study" or (interview* or "Proof of Concept Study" or "brain imaging" or "Experimental Replication" or "focus group*" or "Mathematical Model" or "Scientific Simulation" or "Treatment Outcome" or ((Empirical or Behavior* or Behaviour* or Applied or Population* or Descriptive or Clinical or Field or Followup or Follow-up or Longitudinal or Nonclinical or prospective* or Qualitative or Quantitative or Investigat* or Retrospective or Case or Observation* or twin) adj4 (Study or Studies or Research or Report or Reports or trial*)) or (Grounded adj4 Theor*))).mp. | 8237179 | Advanced |  |  |  |
| 24 | exp "Review Literature as Topic"/ or exp Systematic Review/ or review.pt. or exp "Meta-Analysis as Topic"/ or exp Meta-Analysis/ or Meta-Analysis.pt. or Clinical Protocols/ or guideline.pt. or ((systematic adj2 review) or metaanalys* or meta-analys* or guideline* or protocol*).mp. | 4108077 | Advanced |  |  |  |
| 25 | 23 or 24 | 11140043 | Advanced |  |  |  |
| 26 | 10 and 14 and 15 and 16 and 22 and 25 | 1188 | Advanced |  |  |  |

#### PsycInfo (Ovid)

16/02/2022

**APA PsycInfo <1806 to February Week 1 2022>**

| Search history sorted by search number ascending | | | |  |  |  |
| --- | --- | --- | --- | --- | --- | --- |
| **#** | **Searches** | **Results** | **Type** |  |  |  |
|  | | | | | | |
| 1 | exp Distress/ or exp Stress/ or exp Coping Behavior/ or exp " Resilience (Psychological) "/ or ("common mental disorder*" or CMD or well-being or wellbeing or wellness or burnout or coping or cope or endurance or Hardiness or Resilien* or ((psych* or emotion* or mental or chronic or life or behavio* or social* or interpersonal* or nervous*) adj3 (stress* or distress* or exhaust* or pressure* or shock* or tension*)) or ((dystress or distress) adj3 syndrome*) or (burn adj3 out) or ((occupational or job or work* or professional* or career* or school) adj3 (stress* or distress* or bully* or abuse*)) or ((financ* or Economic*) adj3 (stress* or burden* or hardship* or challenge* or pressure* or toxicit* or strain* or distress* or crisis or crises)) or ((caregiver* or care) adj3 (burden* or exhaust* or strain*)) or ((family or families or familial or home or parent* or maternal* or paternal* or mother* or father*) adj3 (stress* or distress* or crisis or crises or strain*)) or ((moral* or ethic*) adj3 (stress* or distress* or dilemma* or doubt* or paradox* or injur*)) or (critical-incident* adj3 (stress* or distress*)) or ((adapt* or adjust*) adj3 (behavio* or emotion* or psych*))).mp. | 533638 | Advanced |  |  |  |
| 2 | fatigue/ or (((mental* or emotional* or psych* or cogniti* or stress*) adj5 (Fatigue* or exhaust* or Lassitud*)) or (low adj3 alert*)).mp. | 18744 | Advanced |  |  |  |
| 3 | exp Anxiety/ or (Anxiet* or Angst or Hypervigilan* or Nervous* or Anxious* or Catastrophi* or fear or fearing or fears).mp. | 455950 | Advanced |  |  |  |
| 4 | Sadness/ or (Sadness or Unhappiness or sad or unhappy or melanchol*).mp. | 26140 | Advanced |  |  |  |
| 5 | Social Anxiety/ or (social adj2 (avoid* or anxiet* or anxious*)).mp. | 16734 | Advanced |  |  |  |
| 6 | anxiety disorders/ or castration anxiety/ or generalized anxiety disorder/ or panic attack/ or panic disorder/ or separation anxiety disorder/ or trichotillomania/ or exp Neurosis/ or (GAD or ((anxiet* or anxious*) adj3 (disorder* or generalized or generalised)) or (Anxiety adj1 Separation) or cardioneuros* or effort-syndrome or neurosis-cordis or ((cardiac or heart) adj2 (anxiet* or neuros*)) or (heart adj1 (soldier* or complaint or neurogenic)) or (neurocirculat* adj1 (astheni* or dystoni*)) or neuroses or neurosis or neurotic* or Psychoneuro* or panic* or cothymi* or ((anxiety or anxious) adj2 depressi*) or psychastheni* or (asthenic adj1 syndrome) or koro).mp. | 186889 | Advanced |  |  |  |
| 7 | exp "Stress and Trauma Related Disorders"/ or Posttraumatic Growth/ or (psychotrauma* or Trauma or PTSD or DES*NOS or C*PTSD or EPCACE or multitrauma or traumatised or traumatized or DTD or "Enduring Personality Change after Catastrophic Experience*" or (Stress adj3 disorder*) or ((combat or war) adj3 (experience* or disorder* or fatigue or neurosis or neuroses or stress)) or ((Emotional or Complex or chronic or Complicated or Multiple) adj3 Trauma*) or ((acute or military) adj3 Stress) or ((Stress or Crisis) adj3 Reaction*) or ((Post-Traumatic or posttraumatic or Trauma*) adj3 (stress or neurosis or neuroses or syndrome* or Disorder* or psychosis or psychoses or distress* or growth)) or (moral adj2 injury)).mp. | 147404 | Advanced |  |  |  |
| 8 | exp major depression/ or seasonal affective disorder/ or Atypical Depression/ or "Depression (Emotion)"/ or (depression* or depressive or depressed or dysthymia or dysthymic or mdd).mp. | 396623 | Advanced |  |  |  |
| 9 | exp Bipolar Disorder/ or (maniodepressi* or cyclothymi* or (bipolar adj3 (psychos* or depress* or disorder*)) or (depressi* adj3 (mania or manic or mano))).mp. | 50857 | Advanced |  |  |  |
| 10 | 1 or 2 or 3 or 4 or 5 or 6 or 7 or 8 or 9 | 1221066 | Advanced |  |  |  |
| 11 | exp mental health services/ or child guidance clinics/ or community mental health centers/ or exp psychotherapy/ or exp " Psychiatric Hospitals"/ or (Psychotherap* or rehabilitat* or Hypnotherap* or Logotherap* or Psychoanaly* or Psychodrama* or Self-Analy* or Mindful* or CBT or CGT or EMDR or NET or KIDNET or BEPP or psychiatrist* or ((Group or psycho or Hypno or Logo or Mental or ACT or acceptance or aversion or Desensitization or Family or sex or management or Play or Primal or Schema or Couples or Gestalt or Insight or Network or Reality or Bowenian or Conjoint or Exposure or Ego or Feminist or Cognitive or cognition or Narrative or Strategic or Conversion or Persuasion or Affirmative or Existential or Relationship or Multisystemic or Client or Focused or Relational or Behavior* or Behaviour* or Constructivist or Centered or Rational or Emotive or Imagery or Rehearsal or Processing or Reciprocal or Inhibition or Interoceptive or Multiple or Systematic or Trauma*) adj3 Therap*) or ((psycho or self or Dream or Transactional) adj3 analy*) or (psycho adj3 drama) or (Age adj3 Regression) or (Chair adj3 Technique) or (Family adj3 Counseling) or (Guided adj3 Imagery) or (Autogenic adj3 Training) or (Methadone adj3 Maintenance) or (Therapeutic adj3 Community) or (Posthypnotic adj3 Suggestion*) or (Cognitive adj3 Restructuring) or (Contingency adj3 Management) or (Guided adj3 Imagery) or (Motivational adj3 Interviewing) or (Neurolinguistic adj3 Programming) or (Seeking adj3 Safety) or (Self adj3 Psychology) or (Stress adj3 Treatment) or (Traumatic adj3 Reduction) or (Virtual adj3 Exposure) or ((mental or family or trauma*) adj3 (service* or program* or intervention* or care or healthcare or support or healing)) or "safe space" or ((brief or low-intensity) and (CBT or intervention* or therap*)) or (psychiatric adj3 (hospital* or service*))).mp. | 743994 | Advanced |  |  |  |
| 12 | exp Social Services/ or exp Social casework/ or exp Support Groups/ or Social Support/ or Empowerment/ or Self-Care/ or exp animal assisted therapy/ or exp Creative Arts Therapy/ or exp bibliotherapy/ or exp occupational therapy/ or exp " Psychosocial Rehabilitation"/ or Crisis Intervention/ or exp counseling/ or exp virtual reality/ or (psycho-social or Psychosocial or MHPSS or ((intervention* or therap* or support or rehabilit*) adj5 (mental* or psych* or social* or animal or art or dance or music or occupational or recreation*)) or bibliotherap* or psychologist* or Psycho-education* or psychoeducation or PFA or communities or community* or empower* or counsel* or casework* or (Mental adj3 (center* or centre* or program*)) or (social adj3 (work* or psycholog* or service* or therap*)) or (psycholog* adj3 aid)).mp. | 1048694 | Advanced |  |  |  |
| 13 | Preventive Mental Health Services/ or ((prevent* or prophyla* or ((primary or early) adj5 (intervention* or care or service* or treatment))) and (mental* or psych* or social*)).mp. | 189727 | Advanced |  |  |  |
| 14 | 11 or 12 or 13 | 1536215 | Advanced |  |  |  |
| 15 | capacity building/ or (implement* or integrat* or ((scale or scaling or scales) adj1 (up or out or deep)) or (capacit* adj3 build*) or (task* adj3 (share* or sharing))).mp. | 441439 | Advanced |  |  |  |
| 16 | food insecurity/ or food safety/ or Water Safety/ or Hygiene/ or "Conservation (Ecological Behavior)"/ or exp Education/ or exp Housing/ or Urban Planning/ or Community Development/ or exp Social Environments/ or ((water and sanitation and hygiene) or ((WIC or WASH or SNAP) adj2 program*) or ((food or water) adj3 (program* or aid or assistance or insecurit* or securit* or quality or inspect* or ration* or stamp* or healthy or suppl*)) or (nutrion* adj3 (program* or intervention* or assistance)) or protection or (protective adj3 (service* or program*)) or shelter* or ((water or wastewater) adj3 (Decolori* or Purification or softening or treatment)) or ((water or Artesian) adj1 well*) or sanitation or sanitary or latrine* or lavator* or restroom* or toilet* or plumbing or Hygiene or Hygienic or Disinfect* or ((waste or garbage or refuse) adj3 (disposal* or dump* or site* or ground* or manage* or Incinerat*)) or landfill* or education* or training or workshop* or college* or school* or teacher* or teaching or university or universities or (literacy adj1 program*) or ((refugee* or coordination or management) adj1 camp*) or housing or Lodging* or (communit* adj3 (care or network* or develop* or support*)) or ((social or psychosocial or neighbour* or neighbor*) adj3 (care or context* or ecolog* or environment* or integrat* or planning or support*)) or ((city or cities or town* or urban or rural or neighbourhood* or neighborhood*) adj3 (development or plan* or renewal*)) or (development adj3 plan*) or ((relief or aid or rescue) adj2 work*) or (humanitarian adj2 (relief* or response* or agenc* or aid))).mp. | 1746263 | Advanced |  |  |  |
| 17 | (humanitarian adj2 (crisis or crises or setting* or emergenc* or area*)).mp. | 454 | Advanced |  |  |  |
| 18 | exp disasters/ or exp Starvation/ or (disaster* or "critical incident*" or avalanche* or earthquake* or groundshaking* or "mass movement" or Liquefaction* or Volcanic or volcano* or "ash fall" or lahar* or ((pyroclastic or lava) adj1 flow*) or flood* or landslide* or tsunami* or ((tidal or action* or rogue) adj1 wave) or seiche* or typhoon* or cyclone* or hurricane* or storm* or surge or surges or tornado* or wind* or rain* or blizzard* or derecho* or lightening* or thunderstorm* or hail* or sand or sandstorm* or duststorm* or drought* or "extreme temperature*" or "heat wave*" or heatwave* or "cold wave*" or coldwave* or "severe winter condition*" or snow* or ice* or frost* or freeze* or dzud or drought* or fire* or wildfire* or "wild fire*" or landfire* or "land fire*" or (fire* and (brush* or bush* or pasture* or forest*)) or bushfire* or "forest fire*" or (glacial and outburst*) or starvation or famine* or ((natural or Geological or hydrological or meteorological or climatological or Biological or Extraterrestrial or Human-induced or man-made or Technological or Societal) adj1 (accident or accidents or hazard* or crisis or crises or emergenc*)) or "insect infestation*" or grasshopper* or locust* or "foodborne" or "food borne" or (Extraterrestrial and impact) or airburst* or "space weather" or "energetic particles" or "geomagnetic storm*" or shockwave* or "Industrial hazard*" or "chemical spill*" or "gas leak*" or collapse* or explosion* or (industrial and fire*) or "nuclear accident*" or radiation or ((structural or building* or dam or dams or bridge*) adj1 (collapse* or failure*)) or ((transportation or air or road or rail or water) adj1 (accident* or crash*)) or "Air pollution*" or haze or "Power outage*" or "hazardous material*" or ((hazard* or pollution*) adj1 (biological or chemical or radiological)) or "food contamination*" or "financial crisis" or hyperinflation or "currency crisis" or massacre or bomb* or evacuation).mp. | 112838 | Advanced |  |  |  |
| 19 | exp war/ or (war or wars or warring or warfar* or ((armed or zone or area*) adj2 conflict) or ("conflict affected" adj3 (population* or person* or communit*)) or (post adj2 (conflict* or emergenc*)) or "civil unrest" or terrorism or cbrn or cbrne or "chemical, biological, radiological, nuclear and explosive weapons" or "chemical, biological, radiological and nuclear").mp. | 54347 | Advanced |  |  |  |
| 20 | (disaster* adj3 (victim* or affected or survivor*)).mp. | 1447 | Advanced |  |  |  |
| 21 | refugees/ or (refugee* or asylumseek* or IDP* or (asylum adj3 (seek* or political)) or ((forced or irregular) adj3 (migrat* or migrant*)) or (displaced adj3 person*)).mp. | 12222 | Advanced |  |  |  |
| 22 | 17 or 18 or 19 or 20 or 21 | 170084 | Advanced |  |  |  |
| 23 | ("Brain Imaging" or "Clinical Case Study" or "Clinical Trial" or "Empirical Study" or "Experimental Replication" or "Field Study" or "Focus Group" or "Followup Study" or "Interview" or "Longitudinal Study" or "Mathematical Model" or "Nonclinical Case Study" or "Prospective Study" or "Qualitative Study" or "Quantitative Study" or "Retrospective Study" or "Scientific Simulation" or "Treatment Outcome" or "Twin Study" or (interview* or "Proof of Concept Study" or "brain imaging" or "Experimental Replication" or "focus group*" or "Mathematical Model" or "Scientific Simulation" or "Treatment Outcome" or ((Empirical or Behavior* or Behaviour* or Applied or Population* or Descriptive or Clinical or Field or Followup or Follow-up or Longitudinal or Nonclinical or prospective* or Qualitative or Quantitative or Investigat* or Retrospective or Case or Observation* or twin) adj4 (Study or Studies or Research or Report or Reports or trial*)) or (Grounded adj4 Theor*))).mp. | 1374315 | Advanced |  |  |  |
| 24 | exp Systematic Review/ or exp Meta Analysis/ or exp "Treatment Guidelines"/ or (literature review or systematic review or meta analysis).md. or ((systematic adj2 review) or metaanalys* or meta-analys* or guideline* or protocol*).mp. | 328087 | Advanced |  |  |  |
| 25 | 23 or 24 | 1586530 | Advanced |  |  |  |
| 26 | 10 and 14 and 15 and 16 and 22 and 25 | 1141 | Advanced |  |  |  |

#### Embase (Ovid)

16/02/2022

**Embase <1974 to 2022 Week 06>**

| Search history sorted by search number ascending | | | |  |  |  |
| --- | --- | --- | --- | --- | --- | --- |
| **#** | **Searches** | **Results** | **Type** |  |  |  |
|  | | | | | | |
| 1 | exp distress syndrome/ or exp "mental stress"/ or exp psychological adjustment/ or exp coping behavior/ or exp psychological resilience/ or ("common mental disorder*" or CMD or well-being or wellbeing or wellness or burnout or coping or cope or endurance or Hardiness or Resilien* or ((psych* or emotion* or mental or chronic or life or behavio* or social* or interpersonal* or nervous*) adj3 (stress* or distress* or exhaust* or pressure* or shock* or tension*)) or ((dystress or distress) adj3 syndrome*) or (burn adj3 out) or ((occupational or job or work* or professional* or career* or school) adj3 (stress* or distress* or bully* or abuse*)) or ((financ* or Economic*) adj3 (stress* or burden* or hardship* or challenge* or pressure* or toxicit* or strain* or distress* or crisis or crises)) or ((caregiver* or care) adj3 (burden* or exhaust* or strain*)) or ((family or families or familial or home or parent* or maternal* or paternal* or mother* or father*) adj3 (stress* or distress* or crisis or crises or strain*)) or ((moral* or ethic*) adj3 (stress* or distress* or dilemma* or doubt* or paradox* or injur*)) or (critical-incident* adj3 (stress* or distress*)) or ((adapt* or adjust*) adj3 (behavio* or emotion* or psych*))).mp. | 939095 | Advanced |  |  |  |
| 2 | mental fatigue/ or (((mental* or emotional* or psych* or cogniti* or stress*) adj5 (Fatigue* or exhaust* or Lassitud*)) or (low adj3 alert*)).mp. | 25537 | Advanced |  |  |  |
| 3 | exp Anxiety/ or (Anxiet* or Angst or Hypervigilan* or Nervous* or Anxious* or Catastrophi* or fear or fearing or fears).mp. | 1601925 | Advanced |  |  |  |
| 4 | Sadness/ or (Sadness or Unhappiness or sad or unhappy or melanchol*).mp. | 32589 | Advanced |  |  |  |
| 5 | social anxiety/ or (social adj2 (avoid* or anxiet* or anxious*)).mp. | 14900 | Advanced |  |  |  |
| 6 | anxiety disorder/ or acute stress disorder/ or anxiety neurosis/ or cardiac anxiety/ or catastrophizing/ or distress syndrome/ or generalized anxiety disorder/ or koro/ or "mixed anxiety and depression"/ or panic/ or psychasthenia/ or separation anxiety/ or (GAD or ((anxiet* or anxious*) adj3 (disorder* or generalized or generalised)) or (Anxiety adj1 Separation) or cardioneuros* or effort-syndrome or neurosis-cordis or ((cardiac or heart) adj2 (anxiet* or neuros*)) or (heart adj1 (soldier* or complaint or neurogenic)) or (neurocirculat* adj1 (astheni* or dystoni*)) or neuroses or neurosis or neurotic* or Psychoneuro* or panic* or cothymi* or ((anxiety or anxious) adj2 depressi*) or psychastheni* or (asthenic adj1 syndrome) or koro).mp. | 343203 | Advanced |  |  |  |
| 7 | exp posttraumatic stress disorder/ or (psychotrauma* or PTSD or DES*NOS or C*PTSD or EPCACE or DTD or "Enduring Personality Change after Catastrophic Experience*" or (Stress adj3 disorder*) or ((combat or war) adj3 (experience* or disorder* or fatigue or neurosis or neuroses or stress)) or ((Emotional or Complex or chronic or Complicated or Multiple or related) adj3 Trauma*) or ((acute or military) adj3 Stress) or ((Stress or Crisis) adj3 Reaction*) or ((Post-Traumatic or posttraumatic or Trauma*) adj3 (stress or neurosis or neuroses or syndrome* or Disorder* or psychosis or psychoses or distress* or growth)) or (moral adj2 injur*)).mp. | 171216 | Advanced |  |  |  |
| 8 | exp Depression/ or (depression* or depressive or depressed or dysthymia or dysthymic or mdd).mp. | 884348 | Advanced |  |  |  |
| 9 | exp bipolar disorder/ or (maniodepressi* or cyclothymi* or (bipolar adj3 (psychos* or depress* or disorder*)) or (depressi* adj3 (mania or manic or mano))).mp. | 88777 | Advanced |  |  |  |
| 10 | 1 or 2 or 3 or 4 or 5 or 6 or 7 or 8 or 9 | 3056900 | Advanced |  |  |  |
| 11 | exp Mental Health Service/ or psychiatric emergency service/ or exp psychotherapy/ or mental health recovery/ or exp mental hospital/ or (Psychotherap* or rehabilitat* or Hypnotherap* or Logotherap* or Psychoanaly* or Psychodrama* or Self-Analy* or Mindful* or CBT or CGT or EMDR or NET or KIDNET or BEPP or psychiatrist* or ((Group or psycho or Hypno or Logo or Mental or ACT or acceptance or aversion or Desensitization or Family or sex or management or Play or Primal or Schema or Couples or Gestalt or Insight or Network or Reality or Bowenian or Conjoint or Exposure or Ego or Feminist or Cognitive or cognition or Narrative or Strategic or Conversion or Persuasion or Affirmative or Existential or Relationship or Multisystemic or Client or Focused or Relational or Behavior* or Behaviour* or Constructivist or Centered or Rational or Emotive or Imagery or Rehearsal or Processing or Reciprocal or Inhibition or Interoceptive or Multiple or Systematic or Trauma*) adj3 Therap*) or ((psycho or self or Dream or Transactional) adj3 analy*) or (psycho adj3 drama) or (Age adj3 Regression) or (Chair adj3 Technique) or (Family adj3 Counseling) or (Guided adj3 Imagery) or (Autogenic adj3 Training) or (Methadone adj3 Maintenance) or (Therapeutic adj3 Community) or (Posthypnotic adj3 Suggestion*) or (Cognitive adj3 Restructuring) or (Contingency adj3 Management) or (Guided adj3 Imagery) or (Motivational adj3 Interviewing) or (Neurolinguistic adj3 Programming) or (Seeking adj3 Safety) or (Self adj3 Psychology) or (Stress adj3 Treatment) or (Traumatic adj3 Reduction) or (Virtual adj3 Exposure) or ((mental or family or trauma*) adj3 (service* or program* or intervention* or care or healthcare or support or healing)) or "safe space" or ((brief or low-intensity) and (CBT or intervention* or therap*)) or (psychiatric adj3 (hospital* or service*))).mp. | 1416853 | Advanced |  |  |  |
| 12 | exp Social Work/ or exp Social Welfare/ or exp Self Help/ or Social Support/ or Empowerment/ or exp Self Care/ or exp animal assisted therapy/ or exp art therapy/ or exp bibliotherapy/ or dance therapy/ or exp music therapy/ or exp occupational therapy/ or exp recreational therapy/ or exp vocational rehabilitation/ or psychosocial rehabilitation/ or Crisis Intervention/ or Psychosocial Intervention/ or exp Counseling/ or exp virtual reality/ or (psycho-social or Psychosocial or MHPSS or ((intervention* or therap* or support or rehabilit*) adj5 (mental* or psych* or social* or animal or art or dance or music or occupational or recreation*)) or bibliotherap* or psychologist* or Psycho-education* or psychoeducation or PFA or communities or community* or empower* or counsel* or casework* or (Mental adj3 (center* or centre* or program*)) or (social adj3 (work* or psycholog* or service* or therap*)) or (psycholog* adj3 aid)).mp. | 1802223 | Advanced |  |  |  |
| 13 | ((prevent* or prophyla* or ((primary or early) adj5 (intervention* or care or service* or treatment))) and (mental* or psych* or social*)).mp. | 441549 | Advanced |  |  |  |
| 14 | 11 or 12 or 13 | 3083817 | Advanced |  |  |  |
| 15 | capacity building/ or scale up/ or (implement* or integrat* or ((scale or scaling or scales) adj1 (up or out or deep)) or (capacit* adj3 build*) or (task* adj3 (share* or sharing))).mp. | 1485857 | Advanced |  |  |  |
| 16 | exp disaster response/ or Refugee Camp/ or Emergency Shelter/ or exp Food Security/ or exp Food Insecurity/ or exp Food Assistance/ or Water Insecurity/ or exp Water Supply/ or exp Water Quality/ or exp Sanitation/ or exp Hygiene/ or exp Education/ or exp Housing/ or exp city planning/ or exp environmental planning/ or exp Social Environment/ or ((water and sanitation and hygiene) or ((WIC or WASH or SNAP) adj2 program*) or ((food or water) adj3 (program* or aid or assistance or insecurit* or securit* or quality or inspect* or ration* or stamp* or healthy or suppl*)) or (nutrion* adj3 (program* or intervention* or assistance)) or protection or (protective adj3 (service* or program*)) or shelter* or ((water or wastewater) adj3 (Decolori* or Purification or softening or treatment)) or ((water or Artesian) adj1 well*) or sanitation or sanitary or latrine* or lavator* or restroom* or toilet* or plumbing or Hygiene or Hygienic or Disinfect* or ((waste or garbage or refuse) adj3 (disposal* or dump* or site* or ground* or manage* or Incinerat*)) or landfill* or education* or training or workshop* or college* or school* or teacher* or teaching or university or universities or (literacy adj1 program*) or ((refugee* or coordination or management) adj1 camp*) or housing or Lodging* or (communit* adj3 (care or network* or develop* or support*)) or ((social or psychosocial or neighbour* or neighbor*) adj3 (care or context* or ecolog* or environment* or integrat* or planning or support*)) or ((city or cities or town* or urban or rural or neighbourhood* or neighborhood*) adj3 (development or plan* or renewal*)) or (development adj3 plan*) or ((relief or aid or rescue) adj2 work*) or (humanitarian adj2 (relief* or response* or agenc* or aid))).mp. | 5416640 | Advanced |  |  |  |
| 17 | exp humanitarian crisis/ or (humanitarian adj2 (crisis or crises or setting* or emergenc* or area*)).mp. | 1345 | Advanced |  |  |  |
| 18 | exp disaster/ or exp Starvation/ or (disaster* or "critical incident*" or avalanche* or earthquake* or groundshaking* or "mass movement" or Liquefaction* or Volcanic or volcano* or "ash fall" or lahar* or ((pyroclastic or lava) adj1 flow*) or flood* or landslide* or tsunami* or ((tidal or action* or rogue) adj1 wave) or seiche* or typhoon* or cyclone* or hurricane* or storm* or surge or surges or tornado* or wind* or rain* or blizzard* or derecho* or lightening* or thunderstorm* or hail* or sand or sandstorm* or duststorm* or drought* or "extreme temperature*" or "heat wave*" or heatwave* or "cold wave*" or coldwave* or "severe winter condition*" or snow* or ice* or frost* or freeze* or dzud or drought* or fire* or wildfire* or "wild fire*" or landfire* or "land fire*" or (fire* and (brush* or bush* or pasture* or forest*)) or bushfire* or "forest fire*" or (glacial and outburst*) or starvation or famine* or ((natural or Geological or hydrological or meteorological or climatological or Biological or Extraterrestrial or Human-induced or man-made or Technological or Societal) adj1 (accident or accidents or hazard* or crisis or crises or emergenc*)) or "insect infestation*" or grasshopper* or locust* or "foodborne" or "food borne" or (Extraterrestrial and impact) or airburst* or "space weather" or "energetic particles" or "geomagnetic storm*" or shockwave* or "Industrial hazard*" or "chemical spill*" or "gas leak*" or collapse* or explosion* or (industrial and fire*) or "nuclear accident*" or radiation or ((structural or building* or dam or dams or bridge*) adj1 (collapse* or failure*)) or ((transportation or air or road or rail or water) adj1 (accident* or crash*)) or "Air pollution*" or haze or "Power outage*" or "hazardous material*" or ((hazard* or pollution*) adj1 (biological or chemical or radiological)) or "food contamination*" or "financial crisis" or hyperinflation or "currency crisis" or massacre or bomb* or evacuation).mp. | 1924054 | Advanced |  |  |  |
| 19 | military deployment/ or military service/ or exp war/ or exp warfare/ or (war or wars or warring or warfar* or ((armed or zone or area*) adj2 conflict) or ("conflict affected" adj3 (population* or person* or communit*)) or (post adj2 (conflict* or emergenc*)) or "civil unrest" or terrorism or cbrn or cbrne or "chemical, biological, radiological, nuclear and explosive weapons" or "chemical, biological, radiological and nuclear").mp. | 194427 | Advanced |  |  |  |
| 20 | exp Disaster Victim/ or (disaster* adj3 (victim* or affected or survivor*)).mp. | 2563 | Advanced |  |  |  |
| 21 | exp refugee/ or (refugee* or asylumseek* or IDP* or (asylum adj3 (seek* or political)) or ((forced or irregular) adj3 (migrat* or migrant*)) or (displaced adj3 person*)).mp. | 23415 | Advanced |  |  |  |
| 22 | 17 or 18 or 19 or 20 or 21 | 2118041 | Advanced |  |  |  |
| 23 | ("Brain Imaging" or "Clinical Case Study" or "Clinical Trial" or "Empirical Study" or "Experimental Replication" or "Field Study" or "Focus Group" or "Followup Study" or "Interview" or "Longitudinal Study" or "Mathematical Model" or "Nonclinical Case Study" or "Prospective Study" or "Qualitative Study" or "Quantitative Study" or "Retrospective Study" or "Scientific Simulation" or "Treatment Outcome" or "Twin Study" or (interview* or "Proof of Concept Study" or "brain imaging" or "Experimental Replication" or "focus group*" or "Mathematical Model" or "Scientific Simulation" or "Treatment Outcome" or ((Empirical or Behavior* or Behaviour* or Applied or Population* or Descriptive or Clinical or Field or Followup or Follow-up or Longitudinal or Nonclinical or prospective* or Qualitative or Quantitative or Investigat* or Retrospective or Case or Observation* or twin) adj4 (Study or Studies or Research or Report or Reports or trial*)) or (Grounded adj4 Theor*))).mp. | 12482826 | Advanced |  |  |  |
| 24 | Systematic Review/ or "meta analysis (topic)"/ or exp MetaAnalysis/ or exp practice guideline/ or ((systematic adj2 review) or metaanalys* or meta-analys* or guideline* or protocol*).mp. | 2149334 | Advanced |  |  |  |
| 25 | 23 or 24 | 13584393 | Advanced |  |  |  |
| 26 | 10 and 14 and 15 and 16 and 22 and 25 | 1773 | Advanced |  |  |  |

#### Ovid Evidence Based Medicine Reviews

16/02/2022

**All EBM Reviews - Cochrane DSR, ACP Journal Club, DARE, CCA, CCTR, CMR, HTA, and NHSEED**

| Search history sorted by search number ascending | | | |  |  |  |
| --- | --- | --- | --- | --- | --- | --- |
| **#** | **Searches** | **Results** | **Type** |  |  |  |
|  | | | | | | |
| 1 | ("common mental disorder*" or CMD or well-being or wellbeing or wellness or burnout or coping or cope or endurance or Hardiness or Resilien* or ((psych* or emotion* or mental or chronic or life or behavio* or social* or interpersonal* or nervous*) adj3 (stress* or distress* or exhaust* or pressure* or shock* or tension*)) or ((dystress or distress) adj3 syndrome*) or burn-out or ((occupational or job or work* or professional* or career* or school) adj3 (stress* or distress* or bully* or abuse*)) or ((financ* or Economic*) adj3 (stress* or burden* or hardship* or challenge* or pressure* or toxicit* or strain* or distress* or crisis or crises)) or ((caregiver* or care) adj3 (burden* or exhaust* or strain*)) or ((family or families or familial or home or parent* or maternal* or paternal* or mother* or father*) adj3 (stress* or distress* or crisis or crises or strain*)) or ((moral* or ethic*) adj3 (stress* or distress* or dilemma* or doubt* or paradox* or injur*)) or (critical-incident* adj3 (stress* or distress*)) or ((adapt* or adjust*) adj3 (behavio* or emotion* or psych*))).mp. | 314250 | Advanced |  |  |  |
| 2 | (((mental* or emotional* or psych* or cogniti* or stress*) adj5 (Fatigue* or exhaust* or Lassitud*)) or (low adj3 alert*)).mp. | 5404 | Advanced |  |  |  |
| 3 | (Anxiet* or Angst or Hypervigilan* or Nervous* or Anxious* or Catastrophi* or fear or fearing or fears).mp. | 101262 | Advanced |  |  |  |
| 4 | (Sadness or Unhappiness or sad or unhappy or melanchol*).mp. | 3817 | Advanced |  |  |  |
| 5 | (social adj2 (avoid* or anxiet* or anxious*)).mp. | 2906 | Advanced |  |  |  |
| 6 | (GAD or ((anxiet* or anxious*) adj3 (disorder* or generalized or generalised)) or (Anxiety adj1 Separation) or cardioneuros* or effort-syndrome or neurosis-cordis or ((cardiac or heart) adj2 (anxiet* or neuros*)) or (heart adj1 (soldier* or complaint or neurogenic)) or (neurocirculat* adj1 (astheni* or dystoni*)) or neuroses or neurosis or neurotic* or Psychoneuro* or panic* or cothymi* or ((anxiety or anxious) adj2 depressi*) or psychastheni* or (asthenic adj1 syndrome) or koro).mp. | 40344 | Advanced |  |  |  |
| 7 | (psychotrauma* or PTSD or DES*NOS or C*PTSD or EPCACE or DTD or "Enduring Personality Change after Catastrophic Experience*" or (Stress adj3 disorder*) or ((combat or war) adj3 (experience* or disorder* or fatigue or neurosis or neuroses or stress)) or ((Emotional or Complex or chronic or Complicated or Multiple or related) adj3 Trauma*) or ((acute or military) adj3 Stress) or ((Stress or Crisis) adj3 Reaction*) or ((Post-Traumatic or posttraumatic or Trauma*) adj3 (stress or neurosis or neuroses or syndrome* or Disorder* or psychosis or psychoses or distress* or growth)) or (moral adj2 injur*)).mp. | 14756 | Advanced |  |  |  |
| 8 | (depression* or depressive or depressed or dysthymia or dysthymic or mdd).mp. | 103196 | Advanced |  |  |  |
| 9 | (maniodepressi* or cyclothymi* or (bipolar adj3 (psychos* or depress* or disorder*)) or (depressi* adj3 (mania or manic or mano))).mp. | 8447 | Advanced |  |  |  |
| 10 | 1 or 2 or 3 or 4 or 5 or 6 or 7 or 8 or 9 | 442189 | Advanced |  |  |  |
| 11 | (Psychotherap* or rehabilitat* or Hypnotherap* or Logotherap* or Psychoanaly* or Psychodrama* or Self-Analy* or Mindful* or CBT or CGT or EMDR or NET or KIDNET or BEPP or psychiatrist* or ((Group or psycho or Hypno or Logo or Mental or ACT or acceptance or aversion or Desensitization or Family or sex or management or Play or Primal or Schema or Couples or Gestalt or Insight or Network or Reality or Bowenian or Conjoint or Exposure or Ego or Feminist or Cognitive or cognition or Narrative or Strategic or Conversion or Persuasion or Affirmative or Existential or Relationship or Multisystemic or Client or Focused or Relational or Behavior* or Behaviour* or Constructivist or Centered or Rational or Emotive or Imagery or Rehearsal or Processing or Reciprocal or Inhibition or Interoceptive or Multiple or Systematic or Trauma*) adj3 Therap*) or ((psycho or self or Dream or Transactional) adj3 analy*) or (psycho adj3 drama) or (Age adj3 Regression) or (Chair adj3 Technique) or (Family adj3 Counseling) or (Guided adj3 Imagery) or (Autogenic adj3 Training) or (Methadone adj3 Maintenance) or (Therapeutic adj3 Community) or (Posthypnotic adj3 Suggestion*) or (Cognitive adj3 Restructuring) or (Contingency adj3 Management) or (Guided adj3 Imagery) or (Motivational adj3 Interviewing) or (Neurolinguistic adj3 Programming) or (Seeking adj3 Safety) or (Self adj3 Psychology) or (Stress adj3 Treatment) or (Traumatic adj3 Reduction) or (Virtual adj3 Exposure) or ((mental or family or trauma*) adj3 (service* or program* or intervention* or care or healthcare or support or healing)) or "safe space" or ((brief or low-intensity) and (CBT or intervention* or therap*)) or (psychiatric adj3 (hospital* or service*))).mp. | 226554 | Advanced |  |  |  |
| 12 | (psycho-social or Psychosocial or MHPSS or ((intervention* or therap* or support or rehabilit*) adj5 (mental* or psych* or social* or animal or art or dance or music or occupational or recreation*)) or bibliotherap* or psychologist* or Psycho-education* or psychoeducation or PFA or communities or community* or empower* or counsel* or casework* or (Mental adj3 (center* or centre* or program*)) or (social adj3 (work* or psycholog* or service* or therap*)) or (psycholog* adj3 aid)).mp. | 162368 | Advanced |  |  |  |
| 13 | ((prevent* or prophyla* or ((primary or early) adj5 (intervention* or care or service* or treatment))) and (mental* or psych* or social*)).mp. | 52849 | Advanced |  |  |  |
| 14 | 11 or 12 or 13 | 336861 | Advanced |  |  |  |
| 15 | (implement* or integrat* or scale-up or up-scale or scale-out or out-scale or scale-deep or deep-scale or scaling-up or up-scaling or scaling-out or out-scaling or scaling-deep or deep-scaling or scales-up or up-scales or scales-out or out-scales or scales-deep or deep-scales or (capacit* adj3 build*) or (task* adj3 (share* or sharing))).mp. | 309395 | Advanced |  |  |  |
| 16 | ((water and sanitation and hygiene) or ((WIC or WASH or SNAP) adj2 program*) or ((food or water) adj3 (program* or aid or assistance or insecurit* or securit* or quality or inspect* or ration* or stamp* or healthy or suppl*)) or (nutrion* adj3 (program* or intervention* or assistance)) or protection or (protective adj3 (service* or program*)) or shelter* or ((water or wastewater) adj3 (Decolori* or Purification or softening or treatment)) or ((water or Artesian) adj1 well*) or sanitation or sanitary or latrine* or lavator* or restroom* or toilet* or plumbing or Hygiene or Hygienic or Disinfect* or ((waste or garbage or refuse) adj3 (disposal* or dump* or site* or ground* or manage* or Incinerat*)) or landfill* or education* or training or workshop* or college* or school* or teacher* or teaching or university or universities or (literacy adj1 program*) or ((refugee* or coordination or management) adj1 camp*) or housing or Lodging* or (communit* adj3 (care or network* or develop* or support*)) or ((social or psychosocial or neighbour* or neighbor*) adj3 (care or context* or ecolog* or environment* or integrat* or planning or support*)) or ((city or cities or town* or urban or rural or neighbourhood* or neighborhood*) adj3 (development or plan* or renewal*)) or (development adj3 plan*) or ((relief or aid or rescue) adj2 work*) or (humanitarian adj2 (relief* or response* or agenc* or aid))).mp. | 347975 | Advanced |  |  |  |
| 17 | (humanitarian adj2 (crisis or crises or setting* or emergenc* or area*)).mp. | 88 | Advanced |  |  |  |
| 18 | (disaster* or "critical incident*" or avalanche* or earthquake* or groundshaking* or "mass movement" or Liquefaction* or Volcanic or volcano* or "ash fall" or lahar* or ((pyroclastic or lava) adj1 flow*) or flood* or landslide* or tsunami* or ((tidal or action* or rogue) adj1 wave) or seiche* or typhoon* or cyclone* or hurricane* or storm* or surge or surges or tornado* or wind* or rain* or blizzard* or derecho* or lightening* or thunderstorm* or hail* or sand or sandstorm* or duststorm* or drought* or "extreme temperature*" or "heat wave*" or heatwave* or "cold wave*" or coldwave* or "severe winter condition*" or snow* or ice* or frost* or freeze* or dzud or drought* or fire* or wildfire* or "wild fire*" or landfire* or "land fire*" or (fire* and (brush* or bush* or pasture* or forest*)) or bushfire* or "forest fire*" or (glacial and outburst*) or starvation or famine* or ((natural or Geological or hydrological or meteorological or climatological or Biological or Extraterrestrial or Human-induced or man-made or Technological or Societal) adj1 (accident or accidents or hazard* or crisis or crises or emergenc*)) or "insect infestation*" or grasshopper* or locust* or "foodborne" or "food borne" or (Extraterrestrial and impact) or airburst* or "space weather" or "energetic particles" or "geomagnetic storm*" or shockwave* or "Industrial hazard*" or "chemical spill*" or "gas leak*" or collapse* or explosion* or (industrial and fire*) or "nuclear accident*" or radiation or ((structural or building* or dam or dams or bridge*) adj1 (collapse* or failure*)) or ((transportation or air or road or rail or water) adj1 (accident* or crash*)) or "Air pollution*" or haze or "Power outage*" or "hazardous material*" or ((hazard* or pollution*) adj1 (biological or chemical or radiological)) or "food contamination*" or "financial crisis" or hyperinflation or "currency crisis" or massacre or bomb* or evacuation).mp. | 66570 | Advanced |  |  |  |
| 19 | (war or wars or warring or warfar* or ((armed or zone or area*) adj2 conflict) or ("conflict affected" adj3 (population* or person* or communit*)) or (post adj2 (conflict* or emergenc*)) or "civil unrest" or terrorism or cbrn or cbrne or "chemical, biological, radiological, nuclear and explosive weapons" or "chemical, biological, radiological and nuclear").mp. | 8131 | Advanced |  |  |  |
| 20 | (disaster* adj3 (victim* or affected or survivor*)).mp. | 81 | Advanced |  |  |  |
| 21 | (refugee* or asylumseek* or IDP* or (asylum adj3 (seek* or political)) or ((forced or irregular) adj3 (migrat* or migrant*)) or (displaced adj3 person*)).mp. | 886 | Advanced |  |  |  |
| 22 | 17 or 18 or 19 or 20 or 21 | 74999 | Advanced |  |  |  |
| 23 | ("Brain Imaging" or "Clinical Case Study" or "Clinical Trial" or "Empirical Study" or "Experimental Replication" or "Field Study" or "Focus Group" or "Followup Study" or "Interview" or "Longitudinal Study" or "Mathematical Model" or "Nonclinical Case Study" or "Prospective Study" or "Qualitative Study" or "Quantitative Study" or "Retrospective Study" or "Scientific Simulation" or "Treatment Outcome" or "Twin Study" or (interview* or "Proof of Concept Study" or "brain imaging" or "Experimental Replication" or "focus group*" or "Mathematical Model" or "Scientific Simulation" or "Treatment Outcome" or ((Empirical or Behavior* or Behaviour* or Applied or Population* or Descriptive or Clinical or Field or Followup or Follow-up or Longitudinal or Nonclinical or prospective* or Qualitative or Quantitative or Investigat* or Retrospective or Case or Observation* or twin) adj4 (Study or Studies or Research or Report or Reports or trial*)) or (Grounded adj4 Theor*))).mp. | 1136596 | Advanced |  |  |  |
| 24 | ((systematic adj2 review) or metaanalys* or meta-analys* or guideline* or protocol*).mp. | 268437 | Advanced |  |  |  |
| 25 | 23 or 24 | 1218667 | Advanced |  |  |  |
| 26 | 10 and 14 and 15 and 16 and 22 and 25 | 1967 | Advanced |  |  |  |

#### PTSDpubs

16/02/2022

| Set# | Searched for | Databases | Results |
| --- | --- | --- | --- |
| S1 | MAINSUBJECT.EXACT.EXPLODE("Coping Behavior") OR MAINSUBJECT.EXACT("Resilience") OR MAINSUBJECT.EXACT.EXPLODE("Generalized Anxiety Disorder") OR MAINSUBJECT.EXACT.EXPLODE("Panic Disorder") OR MAINSUBJECT.EXACT("Anxiety Disorders") OR MAINSUBJECT.EXACT.EXPLODE("PTSD") OR MAINSUBJECT.EXACT.EXPLODE("Enduring Personality Change") OR MAINSUBJECT.EXACT("(Stress Disorders)") OR MAINSUBJECT.EXACT.EXPLODE("Acute Stress Disorder") OR MAINSUBJECT.EXACT.EXPLODE("Traumatic Neuroses") OR MAINSUBJECT.EXACT.EXPLODE("Mood Disorders") OR TI,AB,SU("common mental disorder*" OR stress OR distress OR mental*-Fatigue* or Anxiet* OR Anxious* OR sadness OR Unhappiness OR (social NEAR/2 (avoid* OR anxiet*)) or ((Post-Traumatic or posttraumatic) NEAR/3 growth) ) | PTSDpubs | 52989 |
| S2 | MAINSUBJECT.EXACT.EXPLODE("Outpatient Treatment") OR MAINSUBJECT.EXACT.EXPLODE("Psychotherapy") OR MAINSUBJECT.EXACT("Psychiatric Hospitals") OR MAINSUBJECT.EXACT("Social Casework") OR MAINSUBJECT.EXACT.EXPLODE("Social Support Networks") OR MAINSUBJECT.EXACT.EXPLODE("Self Help Techniques") OR MAINSUBJECT.EXACT("Animal Assisted Therapy") OR MAINSUBJECT.EXACT.EXPLODE("Creative Arts Therapy") OR MAINSUBJECT.EXACT("Occupational Therapy") OR MAINSUBJECT.EXACT.EXPLODE("Recreation Therapy") OR MAINSUBJECT.EXACT.EXPLODE("Psychosocial Rehabilitation") OR MAINSUBJECT.EXACT("Virtual Reality Exposure") OR TI,AB,SU(psycho-social or Psychosocial or MHPSS ) OR MAINSUBJECT.EXACT.EXPLODE("Prevention") | PTSDpubs | 19822 |
| S3 | TI,AB,SU(implement* or integrat* OR ((scale or scaling or scales) NEAR/1 (up or out or deep)) or (capacit* NEAR/3 build*) OR (task* NEAR/3 (share* OR sharing)) ) | PTSDpubs | 4638 |
| S4 | MAINSUBJECT.EXACT.EXPLODE("Education") OR TI,AB,SU(food OR water OR shelter* OR wastewater OR sanitation OR toilet* OR plumbing OR Hygiene OR waste or garbage OR refugee-camp* OR housing OR communit* OR ((social OR psychosocial) NEAR/3 (care OR integrat* Or support*)) OR ((city OR cities OR town* OR urban OR rural) NEAR/3 (development OR plan* OR renewal*)) OR relief OR aid or rescue OR humanitarian) | PTSDpubs | 13216 |
| S5 | MAINSUBJECT.EXACT.EXPLODE("Disasters") OR MAINSUBJECT.EXACT("Starvation") OR MAINSUBJECT.EXACT.EXPLODE("War") OR MAINSUBJECT.EXACT.EXPLODE("Refugees") OR MAINSUBJECT.EXACT.EXPLODE("Internally Displaced Persons") OR TI,AB,SU(humanitarian NEAR/2 (crisis or crises or setting* or emergenc* or area*) or disaster* ) | PTSDpubs | 20809 |
| S6 | TI,AB,SU( ("treatment outcome" OR "treatment outcomes") or interview* or "Proof of Concept Stud*" or focus-group* or ((Empirical or Behavio* or Applied or Population* or Descriptive or Clinical or Field or Followup or Follow-up or Longitudinal or Nonclinical or prospective* or Qualitative or Quantitative or Investigat* or Retrospective or Case or Observation*) NEAR/4 (Study or Studies or Research or Report or Reports or trial*)) or (Grounded NEAR/4 Theor*)) OR MAINSUBJECT.EXACT("Literature Review") OR MAINSUBJECT.EXACT("Systematic Review") OR MAINSUBJECT.EXACT("Meta Analysis") OR MAINSUBJECT.EXACT("Practice Guideline") | PTSDpubs | 32806 |
| S7 | (MAINSUBJECT.EXACT.EXPLODE("Coping Behavior") OR MAINSUBJECT.EXACT("Resilience") OR MAINSUBJECT.EXACT.EXPLODE("Generalized Anxiety Disorder") OR MAINSUBJECT.EXACT.EXPLODE("Panic Disorder") OR MAINSUBJECT.EXACT("Anxiety Disorders") OR MAINSUBJECT.EXACT.EXPLODE("PTSD") OR MAINSUBJECT.EXACT.EXPLODE("Enduring Personality Change") OR MAINSUBJECT.EXACT("(Stress Disorders)") OR MAINSUBJECT.EXACT.EXPLODE("Acute Stress Disorder") OR MAINSUBJECT.EXACT.EXPLODE("Traumatic Neuroses") OR MAINSUBJECT.EXACT.EXPLODE("Mood Disorders") OR TI,AB,SU("common mental disorder*" OR stress OR distress OR mental*-Fatigue* or Anxiet* OR Anxious* OR sadness OR Unhappiness OR (social NEAR/2 (avoid* OR anxiet*)) or ((Post-Traumatic or posttraumatic) NEAR/3 growth) )) AND (MAINSUBJECT.EXACT.EXPLODE("Outpatient Treatment") OR MAINSUBJECT.EXACT.EXPLODE("Psychotherapy") OR MAINSUBJECT.EXACT("Psychiatric Hospitals") OR MAINSUBJECT.EXACT("Social Casework") OR MAINSUBJECT.EXACT.EXPLODE("Social Support Networks") OR MAINSUBJECT.EXACT.EXPLODE("Self Help Techniques") OR MAINSUBJECT.EXACT("Animal Assisted Therapy") OR MAINSUBJECT.EXACT.EXPLODE("Creative Arts Therapy") OR MAINSUBJECT.EXACT("Occupational Therapy") OR MAINSUBJECT.EXACT.EXPLODE("Recreation Therapy") OR MAINSUBJECT.EXACT.EXPLODE("Psychosocial Rehabilitation") OR MAINSUBJECT.EXACT("Virtual Reality Exposure") OR TI,AB,SU(psycho-social or Psychosocial or MHPSS ) OR MAINSUBJECT.EXACT.EXPLODE("Prevention")) AND TI,AB,SU(implement* or integrat* OR ((scale or scaling or scales) NEAR/1 (up or out or deep)) or (capacit* NEAR/3 build*) OR (task* NEAR/3 (share* OR sharing)) ) AND (MAINSUBJECT.EXACT.EXPLODE("Education") OR TI,AB,SU(food OR water OR shelter* OR wastewater OR sanitation OR toilet* OR plumbing OR Hygiene OR waste or garbage OR refugee-camp* OR housing OR communit* OR ((social OR psychosocial) NEAR/3 (care OR integrat* Or support*)) OR ((city OR cities OR town* OR urban OR rural) NEAR/3 (development OR plan* OR renewal*)) OR relief OR aid or rescue OR humanitarian)) AND (MAINSUBJECT.EXACT.EXPLODE("Disasters") OR MAINSUBJECT.EXACT("Starvation") OR MAINSUBJECT.EXACT.EXPLODE("War") OR MAINSUBJECT.EXACT.EXPLODE("Refugees") OR MAINSUBJECT.EXACT.EXPLODE("Internally Displaced Persons") OR TI,AB,SU(humanitarian NEAR/2 (crisis or crises or setting* or emergenc* or area*) or disaster* )) | PTSDpubs  These databases are searched for part of your query. | 249 |

### Additional file 5: MMAT

| Category of study designs | Methodological quality criteria | Greene (2019) | Greene (2021) | Greene (2022) | Schafer (2014) | Eltayeb (2017) | James (2020) | Weine (2021) | Welton-Mitchelle (2018) |
| --- | --- | --- | --- | --- | --- | --- | --- | --- | --- |
| Screening questions (for all types) | S1. Are there clear research questions? | Yes | Yes | Yes | Yes | Yes | Yes | Yes | Yes |
|  | S2. Do the collected data allow to address the research questions? | Yes | Yes | Yes | Yes | Yes | Yes | Yes | Yes |
| 1. Qualitative | 1.1. Is the qualitative approach appropriate to answer the research question? | N/A | N/A | Yes | Yes | Yes | N/A | N/A | N/A |
|  | 1.2. Are the qualitative data collection methods adequate to address the research question? | N/A | N/A | Yes | Yes | Yes | N/A | N/A | N/A |
|  | 1.3. Are the findings adequately derived from the data? | N/A | N/A | Yes | Yes | Yes | N/A | N/A | N/A |
|  | 1.4. Is the interpretation of results sufficiently substantiated by data? | N/A | N/A | Yes | Yes | Yes | N/A | N/A | N/A |
|  | 1.5. Is there coherence between qualitative data sources, collection, analysis and interpretation? | N/A | N/A | Yes | Yes | Yes | N/A | N/A | N/A |
| 2. Quantitative randomized controlled trials | 2.1. Is randomization appropriately performed? | N/A | Yes | N/A | N/A | N/A | N/A | N/A | N/A |
|  | 2.2. Are the groups comparable at baseline? | N/A | Yes | N/A | N/A | N/A | N/A | N/A | N/A |
|  | 2.3. Are there complete outcome data? | N/A | Yes | N/A | N/A | N/A | N/A | N/A | N/A |
|  | 2.4. Are outcome assessors blinded to the intervention provided? | N/A | Can’t tell | N/A | N/A | N/A | N/A | N/A | N/A |
|  | 2.5 Did the participants adhere to the assigned intervention? | N/A | Yes | N/A | N/A | N/A | N/A | N/A | N/A |
| 3. Quantitative non-randomized | 3.1. Are the participants representative of the target population? | N/A | N/A | N/A | N/A | N/A | N/A | N/A | N/A |
|  | 3.2. Are measurements appropriate regarding both the outcome and intervention (or exposure)? | N/A | N/A | N/A | N/A | N/A | N/A | N/A | N/A |
|  | 3.3. Are there complete outcome data? | N/A | N/A | N/A | N/A | N/A | N/A | N/A | N/A |
|  | 3.4. Are the confounders accounted for in the design and analysis? | N/A | N/A | N/A | N/A | N/A | N/A | N/A | N/A |
|  | 3.5. During the study period, is the intervention administered (or exposure occurred) as intended? | N/A | N/A | N/A | N/A | N/A | N/A | N/A | N/A |
| 4. Quantitative descriptive | 4.1. Is the sampling strategy relevant to address the research question? | N/A | N/A | N/A | N/A | N/A | N/A | N/A | N/A |
|  | 4.2. Is the sample representative of the target population? | N/A | N/A | N/A | N/A | N/A | N/A | N/A | N/A |
|  | 4.3. Are the measurements appropriate? | N/A | N/A | N/A | N/A | N/A | N/A | N/A | N/A |
|  | 4.4. Is the risk of nonresponse bias low? | N/A | N/A | N/A | N/A | N/A | N/A | N/A | N/A |
|  | 4.5. Is the statistical analysis appropriate to answer the research question? | N/A | N/A | N/A | N/A | N/A | N/A | N/A | N/A |
| 5. Mixed methods | 5.1. Is there an adequate rationale for using a mixed methods design to address the research question? | Yes | N/A | N/A | N/A | N/A | Yes | Yes | Yes |
|  | 5.2. Are the different components of the study effectively integrated to answer the research question? | Yes | N/A | N/A | N/A | N/A | Yes | Yes | Yes |
|  | 5.3. Are the outputs of the integration of qualitative and quantitative components adequately interpreted? | Yes | N/A | N/A | N/A | N/A | Yes | Yes | Yes |
|  | 5.4. Are divergences and inconsistencies between quantitative and qualitative results adequately addressed? | Yes | N/A | N/A | N/A | N/A | Can’t tell | Can’t tell | Can’t tell |
|  | 5.5. Do the different components of the study adhere to the quality criteria of each tradition of the methods involved? | Yes | N/A | N/A | N/A | N/A | Can’t tell | Can’t tell | Can’t tell |
